# Ventricular Arrhythmia and Sudden Death Risk With Concomitant Antipsychotic and SSRI Use

**DOI:** 10.1001/jamanetworkopen.2026.6028

**Published:** 2026-04-09

**Authors:** Hsiu-Ting Chien, Shu-Wen Lin, Te-Jung Kung, Chi-Chuan Wang, Yaa-Hui Dong, Tzung-Jeng Hwang, Fang-Ju Lin, Sengwee Toh

**Affiliations:** 1 Graduate Institute of Clinical Pharmacy, College of Medicine, National Taiwan University, Taipei, Taiwan; 2 Department of Population Medicine, Harvard Medical School and Harvard Pilgrim Health Care Institute, Boston, Massachusetts; 3 School of Pharmacy, College of Medicine, National Taiwan University, Taipei, Taiwan; 4 Department of Pharmacy, National Taiwan University Hospital, Taipei, Taiwan; 5 Department of Pharmacy, College of Pharmaceutical Sciences, National Yang Ming Chiao Tung University, Taipei, Taiwan; 6 Institute of Public Health, School of Medicine, National Yang Ming Chiao Tung University, Taipei, Taiwan; 7 Department of Psychiatry, College of Medicine and National Taiwan University Hospital, National Taiwan University, Taipei, Taiwan; 8 Department of Pharmacy, National Taiwan University Cancer Center, Taipei, Taiwan

## Abstract

**Importance:**

Antipsychotics and selective serotonin reuptake inhibitors (SSRIs) are each associated with increased risk of ventricular arrhythmia or sudden death. Their concurrent use may further elevate this risk; however, clinical evidence remains limited.

**Objective:**

To evaluate the risk of ventricular arrhythmia or sudden death in patients concurrently prescribed an antipsychotic and an SSRI.

**Design, Setting, and Participants:**

This cohort study used a sequential target trial emulation method to analyze insurance claims data from health insurance databases in the US (2010-2023) and Taiwan (2010-2021). Eligible patients (adults initiating an antipsychotic with no SSRI use in the prior year) were included and assessed weekly for SSRI initiation from weeks 1 to 52 following antipsychotic initiation. Fifty-two weekly trials were emulated to assess the 1-year ventricular arrhythmia or sudden death risk following SSRI initiation in patients newly treated with antipsychotics. A per-protocol approach with inverse probability weighting and adjustment for time-varying covariates was applied.

**Exposures:**

SSRI initiation within 52 weeks after antipsychotic initiation, with patients classified weekly as SSRI initiators or noninitiators.

**Main Outcomes and Measures:**

The outcome of interest was incident ventricular arrhythmia or sudden death. Hazard ratios (HRs) with 95% CIs were estimated using weighted pooled logistic regression models with robust variance estimators.

**Results:**

Overall, 307 818 unique patients in the US cohort (4 370 289 person-trials; mean [SD] age, 46.6 [18.8] years; 2 764 180 female [61.2%]) and 191 080 unique patients in the Taiwan cohort (2 150 639 person-trials; mean [SD] age, 51.0 [18.3] years; 1 184 464 female [55.1%]) met the eligibility criteria. In the adjusted cohorts, 52 825 US patients (17.2%) and 25 203 Taiwanese patients (14.7%) initiated SSRIs. The adjusted HRs for concurrent antipsychotic and SSRI use vs antipsychotic alone were 1.51 (95% CI, 1.04-2.19) in the US and 3.32 (95% CI, 2.26-4.88) in Taiwan. For high-risk SSRIs, citalopram and escitalopram, the adjusted HRs were 2.20 (95% CI, 1.39-3.46) and 2.84 (95% CI, 1.75-4.62), respectively. Sensitivity analyses supported robustness of primary findings, and positive and negative control analyses suggested limited residual confounding.

**Conclusions and Relevance:**

In this cohort study of adults initiating antipsychotics in the US and Taiwan, SSRI initiation in antipsychotic users was associated with an increased risk of ventricular arrhythmia or sudden death. Although rare, the potential severity of these events supported the need for cautious use of this combination.

## Key Points

**Question:**

Among patients initiating antipsychotics, is subsequent initiation of selective serotonin reuptake inhibitors (SSRIs) associated with an increased risk of ventricular arrhythmia or sudden death?

**Findings:**

In this cohort study using sequential target trial emulation to analyze claims data of 307 818 US and 171 337 Taiwanese adults, concurrent use of antipsychotics and SSRIs was associated with an increased risk of ventricular arrhythmia or sudden death compared with antipsychotic use alone. The association was observed in both data sources.

**Meaning:**

These results suggest that although ventricular arrhythmia or sudden death events were rare, their potential severity underscores the importance of cautious prescribing and monitoring when SSRIs are initiated in patients receiving antipsychotics.

## Teaser

This cohort study evaluates the association of concurrent antipsychotic and selective serotonin reuptake inhibitor (SSRI) use with risk of ventricular arrhythmia or sudden death among adults in the US and Taiwan.

## Introduction

Medication-related ventricular arrhythmia (VA) and sudden death are rare but serious safety concerns.^1^ Antipsychotics and antidepressants can inhibit the rapid delayed rectifier potassium current, leading to QT prolongation.^2^ This electrophysiologic disturbance can trigger torsades de pointes (TdP), a type of VA, which may ultimately result in sudden death.^3,4^

Although both drug classes can prolong the QTc,^5,6^ evidence for clinical VA and sudden death differs. For antipsychotics, evidence is relatively consistent: both first- and second-generation agents have been associated with higher sudden death rates compared with nonuse.^7,8^ In contrast, evidence for antidepressants is mixed: a case-time-control study found a modest increase in out-of-hospital cardiac arrest (OHCA) with tricyclic antidepressants (TCAs) and selective serotonin reuptake inhibitors (SSRIs), particularly for citalopram, whereas serotonin-norepinephrine reuptake inhibitors (SNRIs) showed no association.^9^ A cohort study found no increased VA or sudden death risk with SSRIs even at high doses, leaving the SSRI-related risk uncertain.^10^

Despite uncertainty surrounding SSRI-associated cardiac risk, their concurrent use with antipsychotics remains common in clinical practice. In nonpsychotic depression, antidepressants are typically initiated first, with antipsychotics added for augmentation when needed.^11^ In schizophrenia, antipsychotics are the foundational treatment, and antidepressants may be added for depressive symptoms.^12^ For psychotic depression, guidelines recommend combination therapy with antipsychotics and antidepressants.^13,14^

While concurrent use can be clinically beneficial, concerns about drug-drug interactions that could increase arrhythmias remain. Both drug classes can inhibit delayed rectifier potassium current and may enhance the effects of other QT-prolonging drugs.^15^ An in vitro study suggests that certain combinations may exhibit synergistic rather than additive effects.^16^ Pharmacokinetic interactions may additionally increase exposure to QT-prolonging drugs. For instance, plasma concentrations of the antipsychotic risperidone, a CYP2D6 substrate, can be elevated when coadministered with the SSRI fluoxetine, a strong CYP2D6 inhibitor.^17^ A 75% increase in the active form of risperidone when coadministered with fluoxetine has been reported.^18^ Such increases in QT-prolonging drug exposure may raise the risk of medication-induced VA or sudden death.^7^

The clinical impact of these interactions remains uncertain. Nearly all prior studies evaluated antipsychotics and SSRIs separately, and evidence on arrhythmic outcomes with concomitant use remains limited. To address these uncertainties, we used 2 large insurance claims databases to emulate a sequential target trial and estimate VA or sudden death risk following SSRI initiation among antipsychotic users.

## Methods

### Data Source

This study used the US MarketScan Research Databases (Merative LP) (January 1, 2010, to December 31, 2023) and Taiwan’s National Health Insurance Research Database (NHIRD) (January 1, 2010, to December 31, 2021). This study was reviewed and approved by the institutional review boards of Harvard Pilgrim Health Care and National Taiwan University Hospital and is reported in accordance with the Strengthening the Reporting of Observational Studies in Epidemiology (STROBE) reporting guideline for cohort studies. The requirement for informed consent was waived due to the use of deidentified data and the retrospective design of the study.

MarketScan and NHIRD are longitudinal claims databases that capture medically attended events. MarketScan contains inpatient and outpatient claims and outpatient dispensing, covering approximately 166 million individuals enrolled in commercial or Medicare supplemental insurance across the US.^19^ NHIRD provides near-universal coverage of the 23 million population of Taiwan, including demographics, health care encounters, diagnoses, procedures, and prescription dispensing.^20^ Mortality data were available through December 31, 2015, in MarketScan and throughout the study period in NHIRD. Diagnoses were coded using the *International Classification of Diseases, Ninth Revision, Clinical Modification* (*ICD-9-CM*) until September 30, 2015, for the US cohort and December 31, 2015, for the Taiwanese cohort, and with *International Statistical Classification of Diseases and Related Health Problems, Tenth Revision (ICD-10)* thereafter. Because practice patterns and medication selection may vary across health care systems, and reimbursement policies and population characteristics may also differ (eg, CYP polymorphisms), we analyzed the 2 cohorts and present their results separately.

### Study Design, Population, and Setting

We first conceptualized a hypothetical randomized trial. The trial enrolled adults (aged 18 years or older) with psychotic disorders newly initiating outpatient antipsychotics, excluding those with prior antipsychotic or SSRI use and those with VA or sudden death during the 365-day washout. While remaining on treatment, participants were randomly assigned to add an SSRI (treatment) or not (control). Follow-up began at randomization and ended at VA or sudden death, deviation from the assigned group (per-protocol [PP] approach), deaths not fitting the definition of sudden death, discontinuation of the index antipsychotic, study end, or 52 weeks after enrollment, whichever occurred first (eTable 1 in the Supplement 1).

**Figure 1.**
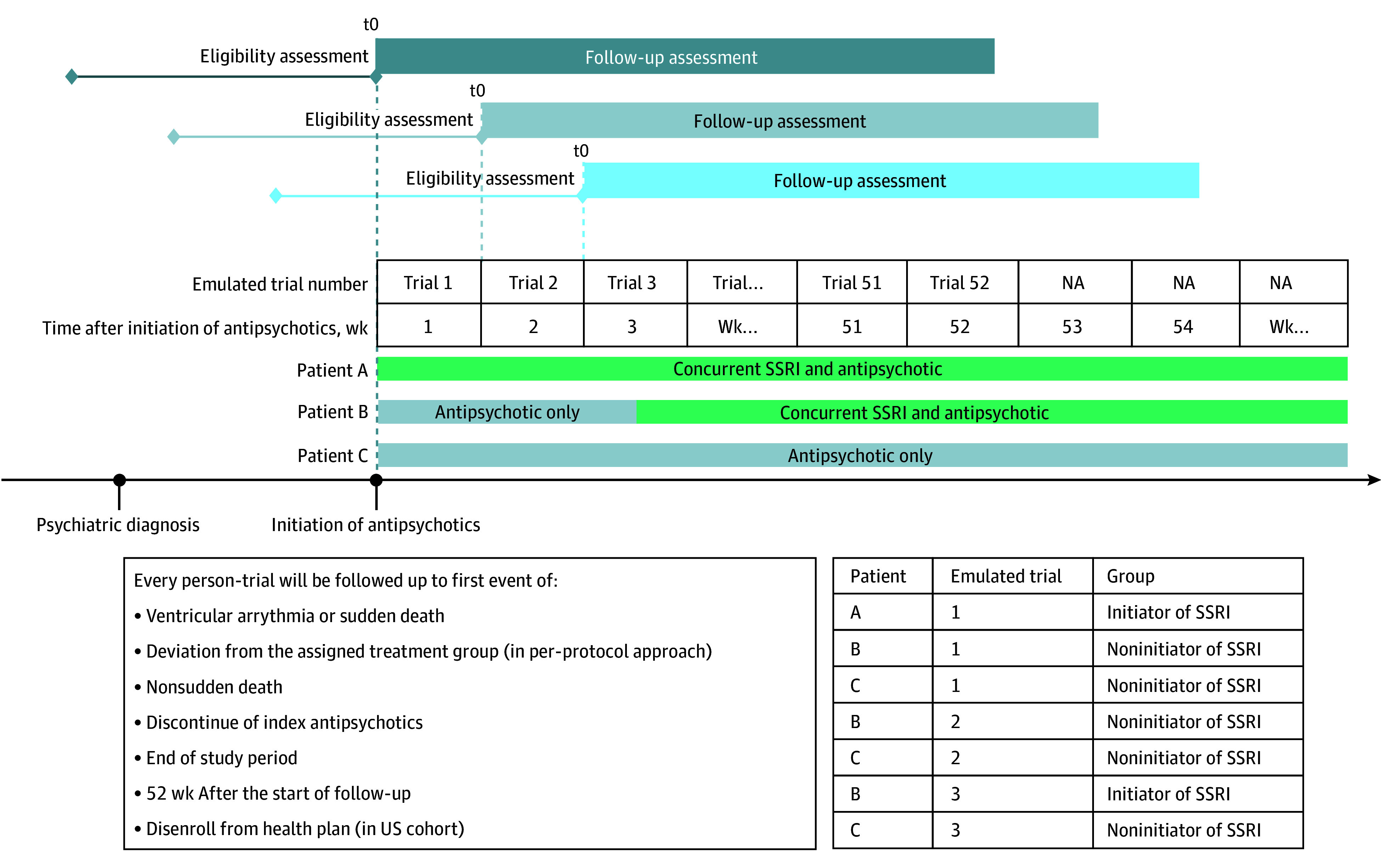
Study Design Diagram of Sequential Weekly Target Trials At the beginning of each week (t_0_) from week 1 through week 52 after antipsychotic initiation, eligibility for trial was reassessed. Individuals who have not yet initiated a selective serotonin reuptake inhibitor (SSRI) and remain on continuous antipsychotic therapy are eligible to enter or reenter the target trial for that week. Within each weekly person-trial, participants are classified as SSRI initiators if they begin SSRI treatment during the 7-day treatment assessment window (days zero to 6 from the weekly index date); otherwise, they are classified as noninitiators. Follow-up continues until the earliest occurrence of: VA or sudden death, nonsudden death, discontinuation of the index antipsychotic, 52 weeks following the trial-specific index date, end of study period, or health plan disenrollment (US cohort only). The diagram illustrates how 3 example patients (A, B, and C) could participate in multiple weekly trials from antipsychotic initiation onward, and how their classification as SSRI initiators or noninitiators varies over time based on when SSRI treatment is started.

Given the challenge of identifying time zero for SSRI noninitiators, we emulated the target trial via 52 weekly trials after antipsychotic initiation (Figure 1).^21,22^ Eligibility criteria were consistent across weekly trials: age 18 years or older, any recorded psychotic disorder diagnosis, no prior SSRI use, and no history of VA or sudden death. Individuals who continued receiving antipsychotics and had not started an SSRI could enter the following weekly trials. For the US cohort, at least 1 year of continuous insurance enrollment prior to antipsychotic initiation was required. This requirement was not applicable for the Taiwan cohort due to universal health care coverage.

In each weekly trial, individuals were classified into 2 strategies: (1) SSRI initiation within that week or (2) no SSRI initiation. Follow-up began at day zero of the trial week and continued until the earliest occurrence of: VA or sudden death not qualifying as sudden death, deviation from the assigned group, discontinuation of the index antipsychotic, December 31, 2023 (for the US cohort), December 31, 2021 (for the Taiwan cohort), 52 weeks of follow-up, or end of insurance enrollment (US cohort only). Medication discontinuation was defined as a gap of more than 30 days without a refill after the last prescription, with the discontinuation date defined as 30 days after the last prescription. VA and sudden death were identified using validated algorithms based on primary inpatient or emergency department (ED) diagnoses (*ICD-9-CM*/*ICD-10-CM* codes 427.1, 427.4, 427.5, 798.x, I46.x, I47.0, I47.2, I49.0, and R99; positive predictive value of 85.3% in the US cohort and between 92.1% and 96.4% in the Taiwan cohort in validation studies).^23,24^ In the US cohort, all ED diagnoses were included due to the lack of information on diagnosis position in MarketScan.

### Covariates

Covariates included demographics, comorbidities, concomitant QT-prolonging drugs and CYP inhibitors, and preindex health care resource utilization (HCRU), assessed during the 365 days preceding day zero (baseline) of each person-trial (eFigure 1 in Supplement 1). Comorbidities were identified using 2 or more outpatient or ER diagnoses or 1 or more inpatient diagnosis, covering all Charlson comorbidities plus cardiovascular and mental disorders. TdP-related acute conditions, including aortic stenosis, septic shock, and Takotsubo cardiomyopathy, were captured via inpatient diagnoses within 30 days. Medication use was determined from outpatient dispensing (both databases) and inpatient claims (NHIRD only) within 90 days. HCRU included hospitalizations, outpatient visits, and ED encounters prior to baseline.

### Statistical Analysis

Baseline characteristics were summarized as totals or mean values. To address confounding from nonrandomized SSRI initiation, we applied stabilized inverse probability of treatment weighting (IPTW) based on a logistic regression model, with weights truncated at the first and 99th percentiles.^25^ Covariate balance was assessed using standardized mean differences (absolute values below 0.1).^26^

To address potential selection bias from differential censoring in the PP analysis, we used inverse probability of censoring weighting (IPCW) to account for deviations from the assigned SSRI strategy, discontinuation of the index antipsychotic, and loss to follow-up (end of insurance enrollment in the US cohort), adjusting for the following time-varying covariates: TdP-related acute conditions, concomitant medications, and HCRU in the prior 30 days. Outcomes were compared using pooled logistic regression to approximate hazard ratios (HRs).^22,27^ Given that individuals could participate in multiple trials, we applied robust variance estimation to obtain conservative 95% CIs.^28^

To examine risk by SSRI type, we stratified analyses by the CredibleMeds classification of TdP risk: known risk (TdP documented under standard use; citalopram, escitalopram) and conditional risk (TdP observed only under certain conditions; fluoxetine, paroxetine, fluvoxamine, sertraline, vilazodone, vortioxetine). Subgroup analyses were conducted by sex, age group (below age 65 years vs 65 years or older), antipsychotic CYP2D6 substrate status, TdP risk (known vs not known), and baseline depression. Sensitivity analyses varied the discontinuation window (90 days) and maximum follow-up (26 or 104 weeks); excluded R99-coded outcomes (to address potential overestimation); omitted baseline depression adjustment (to avoid adjusting for a potential mediator); and restricted the MarketScan analysis to pre-2016 data, when mortality data were more complete. An intention-to-treat (ITT) analysis extended follow-up regardless of treatment deviations.

To examine the potential impact of unmeasured confounders, analyses were repeated using SNRIs as a negative control exposure (similar indications, no observed OHCA association), TCAs as a positive control exposure (similar indications, reported higher OHCA risk), and carditis as a negative control outcome (acute cardiac diagnosis that may share unmeasured factors but is not plausibly related to SSRIs).^9^ All analyses were conducted using SAS version 9.4 (SAS Institute Inc). The threshold for significance was *P* < .05 in 2-sided tests.

## Results

### Patient Enrollment and Baseline Characteristics

A total of 307 818 patients in the US cohort (4 370 289 person-trials; mean [SD] age, 46.6 [18.8] years; 2 764 180 female [61.2%]) and 191 080 in the Taiwan cohort (2 150 639 person-trials; mean [SD] age, 51.0 [18.3] years; 1 184 464 female [55.1%]) met the eligibility criteria. Balance was achieved in both cohorts after IPTW, while the Taiwan cohort required an additional 2.5% trimming of extreme weights,^29^ resulting in 171 337 patients for analysis. After IPTW, baseline covariate distributions were standardized to the overall eligible cohort (trimming in Taiwan emphasized the covariate-overlap region). The mean (SD) age was 46.7 (18.8) years in the US and 51.1 (18.3) years in Taiwan. Diagnosis of depression was more common in Taiwan than in the US (1 019 535 [51.0%] vs 666 235 [15.2%]) (Table 1; eTable 2 in Supplement 1). SSRI initiation within 52 weeks following initial antipsychotic dispensing occurred among 52 825 patients (17.2%) in the US cohort and 25 203 patients (14.7%) in the Taiwan cohort. Allowing noninitiators to contribute to multiple person-trials until SSRI initiation or censoring yielded 52 825 initiator and 4 317 464 noninitiator person-trials (US cohort) and 25 203 initiator and 1 974 404 noninitiator person-trials (Taiwan cohort) (eFigure 2 in Supplement 1).

**Table 1.** Weighted Baseline Characteristics of SSRI Initiators and Noninitiators at the Start of Trial Follow-Up in US and Taiwan Cohorts

Characteristics	US cohort			Taiwan cohort		
	Initiators, No. (%) (n = 53 699)^a^	Noninitiators, No. (%) (n = 4 317 456)^a^	SMD	Initiators, No. (%) (n = 31 915)^a^	Noninitiators, No. (%) (n = 1 967 692)^a^	SMD
Age, mean (SD), y	46.3 (19.0)	46.7 (18.8)	−0.02	51.8 (18.8)	51.1 (18.3)	0.04
Sex						
Female	32 262 (60.1)	2 641 838 (61.2)	−0.02	17 409 (54.5)	1 073 587 (54.6)	0.00
Male	21 437 (39.9)	1 675 618 (38.8)		14 506 (45.5)	894 105 (45.4)	
CCI score						
0	49 845 (92.8)	4 025 566 (93.2)	0.02	17 977 (56.3)	1 176 743 (59.8)	0.08
1	2386 (4.4)	187 213 (4.3)		6234 (19.5)	368 000 (18.7)	
2	1361 (2.5)	97 732 (2.3)		3556 (11.1)	204 297 (10.4)	
≥3	106 (0.2)	6945 (0.2)		4148 (13.0)	218 652 (11.1)	
Cardiometabolic comorbidities						
Atrial fibrillation	48 (0.1)	3673 (0.1)	0.00	372 (1.2)	19 807 (1.0)	0.02
Coronary artery disease	82 (0.2)	6965 (0.2)	0.00	2545 (8.0)	136 708 (6.9)	0.04
Diabetes	1719 (3.2)	134 010 (3.1)	0.01	4527 (14.2)	257 058 (13.1)	0.03
Hyperlipidemia	1251 (2.3)	99 984 (2.3)	0.00	5166 (16.2)	284 286 (14.4)	0.05
Hypertension	496 (0.9)	45 872 (1.1)	−0.01	9015 (28.2)	507 226 (25.8)	0.06
Valvular heart disease	39 (0.1)	3749 (0.1)	−0.01	463 (1.5)	24 277 (1.2)	0.02
Comorbidities associated with TdP						
AV block	0	52 (<0.1)	0.00	25 (0.1)	591 (<0.1)	0.02
Hyperparathyroidism	13 (<0.1)	621 (<0.1)	0.01	41 (0.1)	1476 (0.1)	0.02
Hypothyroidism	1161 (2.2)	93 477 (2.2)	0.00	321 (1.0)	20 947 (1.1)	−0.01
Rheumatic arthritis	4 (<0.1)	805 (<0.1)	−0.01	281 (0.9)	13 818 (0.7)	0.02
Psychiatric comorbidities						
Alcohol use disorder	1156 (2.2)	92 910 (2.2)	0	1332 (4.2)	68 661 (3.5)	0.04
Anxiety	653 (1.2)	57 341 (1.3)	−0.01	12 234 (38.3)	693 762 (35.3)	0.06
Depression	7036 (13.1)	659 199 (15.3)	−0.06	16 542 (51.8)	1 002 993 (51.0)	0.02
Schizophrenia	960 (1.8)	70 660 (1.6)	0.01	3908 (12.2)	272 186 (13.8)	−0.05
Comedications						
Medication with known risk of QT prolongation	5423 (10.1)	435 262 (10.1)	0.00	5574 (17.5)	302 761 (15.4)	0.06
Medications as strong CYP inhibitors						
CYP1A2 inhibitors	610 (1.1)	50 681 (1.2)	0.00	336 (1.1)	16 419 (0.8)	0.02
CYP2D6 inhibitors	6043 (11.3)	439 498 (10.2)	0.03	2319 (7.3)	96 741 (4.9)	0.1
CYP3A4 inhibitors	161 (0.3)	12 228 (0.3)	0.00	463 (1.5)	25 778 (1.3)	0.01
Other antidepressants	18 909 (35.2)	1 540 819 (35.7)	−0.01	13 301 (41.7)	739 098 (37.6)	0.08
HCRU in prior 1 y, mean (SD)						
OPD visits	21.5 (20.3)	21.3 (19.6)	0.01	23.0 (18.0)	21.7 (17.0)	0.07
ED visits	1.1 (2.2)	1.0 (2.0)	0.04	0.7 (1.5)	0.6 (1.4)	0.07
Total hospitalization stays	3.9 ± 11.5	3.4 (9.4)	0.04	3.3 (15.0)	3.0 (15.3)	0.02

Abbreviations: AV block, atrioventricular block; CCI, Charlson comorbidity index; CYP, cytochrome P450; ED, emergency department; HCRU, health care resource utilization; OPD, outpatient department; SMD, standardized mean difference; SSRI, selective serotonin reuptake inhibitor; TdP, torsades de pointes.

^a^ Totals represent the number of person-trials; individuals could contribute to multiple noninitiator trials until SSRI initiation or censoring.

### Associations Between Concomitant Use of Antipsychotics and SSRIs and VA or Sudden Death

Across 52 emulated sequential target trials, VA or sudden death was rare. In the US cohort, VA or sudden death occurred in 39 SSRI initiators (0.1%) and 2666 SSRI noninitiators (0.1%), an absolute difference of approximately 1 per 10 000 person-trials. In Taiwan, VA or sudden death occurred in 47 initiators (0.2%) and 3155 noninitiators (0.2%), an absolute difference of approximately 3 per 10 000 person-trials. The adjusted HR for VA or sudden death associated with SSRI initiation was 1.51 (95% CI, 1.04-2.19) in the US cohort and 3.32 (95% CI, 2.26-4.88) in the Taiwan cohort (Table 2). In the US cohort, known-risk SSRIs were associated with a higher risk of VA or sudden death (HR, 2.20; 95% CI, 1.39-3.46), while conditional-risk SSRIs had no significant association (HR, 0.95; 95% CI, 0.56-1.62). In Taiwan, risk was elevated for both known-risk (HR, 2.84; 95% CI, 1.75-4.62) and conditional-risk SSRIs (HR, 2.85; 95% CI, 1.79-4.54); the conditional-risk association appeared driven by sertraline (HR, 4.16; 95% CI, 2.47-7.00).

**Table 2.** Association Between Concomitant SSRI Use and VA or Sudden Death Among Patients Receiving Antipsychotics

Risk category^a^	Per-protocol analysis^b^				Intention-to-treat analysis^c^			
	No. of events/initiators	No. of events/all individuals	Adjusted HR (95% CI)	*P* value	No. of events/initiators	No. of events/all individuals	Adjusted HR (95% CI)	*P* value
**US cohort**								
Total cohort	39/52 825	2705/4 370 289	1.51 (1.04-2.19)	.03	39/52 825	2922/4 370 289	1.28 (0.91-1.81)	.16
Subset for known-risk SSRIs	23/20 915	2689/4 173 480	2.20 (1.39-3.46)	.001	23/20 915	2832/4 173 480	1.85 (1.19-2.86)	.006
Subset for conditional-risk SSRIs	16/31 910	2682/4 255 518	0.95 (0.56-1.62)	.85	16/31 910	2756/4 255 518	0.87 (0.52-1.46)	.61
**Taiwan cohort** ^d^								
Total cohort	47/25 203	3202/1 999 607	3.32 (2.26-4.88)	<.001	55/25 203	3427/1 999 607	2.00 (1.47-2.72)	<.001
Subset for known-risk SSRIs	24/12 388	3179/1 936 166	2.84 (1.75-4.62)	<.001	26/12 388	3289/1 936 166	1.87 (1.23-2.84)	.003
Subset for conditional-risk SSRIs	23/12 569	3199/1 949 558	2.85 (1.79-4.54)	<.001	29/12 569	3314/1 949 558	2.10 (1.41-3.11)	<.001

Abbreviations: HR, hazard ratio; SSRI, selective serotonin reuptake inhibitor; VA, ventricular arrhythmia.

^a^ Known and conditional risk refers to SSRIs categorized by CredibleMeds based on their potential to cause torsades de pointes. Known-risk SSRIs include citalopram and escitalopram. Conditional-risk SSRIs include fluoxetine, paroxetine, fluvoxamine, sertraline, vilazodone, and vortioxetine.

^b^ In the per-protocol analysis, patients who deviate from the initial treatment in a given person-trial are censored at the time of deviation. HRs are estimated using inverse probability weighting, which accounts for baseline covariates and time-varying covariates related to treatment deviations and censoring.

^c^ Adjusted HRs for the intention-to-treat analysis are estimated using inverse probability of treatment weighting based on baseline covariates.

^d^ The Taiwan cohort excludes patients with a propensity score outside the 2.5th and 97.5th percentiles, resulting in slightly smaller sample sizes compared with those shown in eFigure 2 in Supplement 1.

The ITT analyses yielded estimates closer to the null, particularly in the US (HR, 1.28; 95% CI, 0.91-1.81). The estimate in Taiwan remained elevated (HR, 2.00; 95% CI, 1.47-2.72).

### Subgroup and Sensitivity Analyses

**Figure 2.**
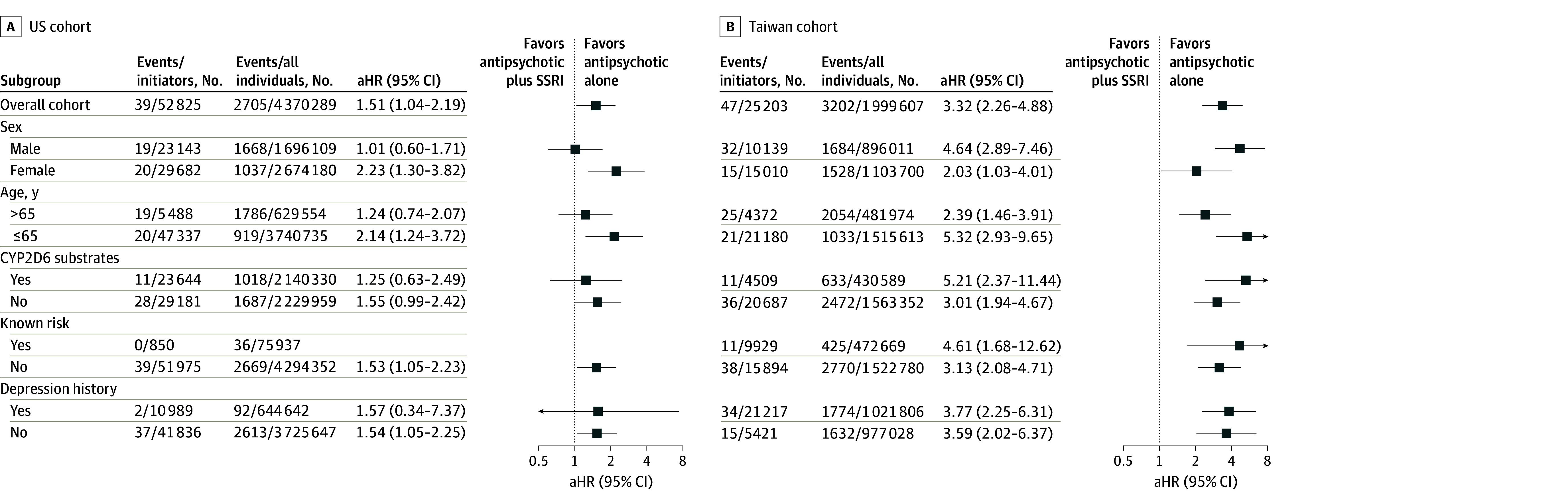
Per-Protocol Analysis of the Association Between Concomitant SSRI Use and VA or Sudden Death in Patients Receiving Antipsychotics (Subgroup Analyses) Age was defined as the age at the time of cohort entry (antipsychotic initiation). An antipsychotic was classified as a CYP2D6 substrate if it is primarily metabolized by the cytochrome P450 2D6 enzyme. Known risk refers to antipsychotics categorized by CredibleMeds as having a known risk of torsades de pointes. Depression history was defined as at least two outpatient diagnoses or one inpatient diagnosis within 365 days prior to the date of antipsychotic initiation. Adjusted HRs were estimated using inverse probability weighting to account for baseline covariates and time-varying covariates related to treatment deviations and censoring. Patients with propensity scores outside the 2.5th and 97.5th percentiles were excluded from the Taiwan cohort to reduce potential confounding. CYP2D6 indicates cytochrome P450 2D6; HR, hazard ratio; SSRI, selective serotonin reuptake inhibitor; VA, ventricular arrhythmia.

**Figure 3.**
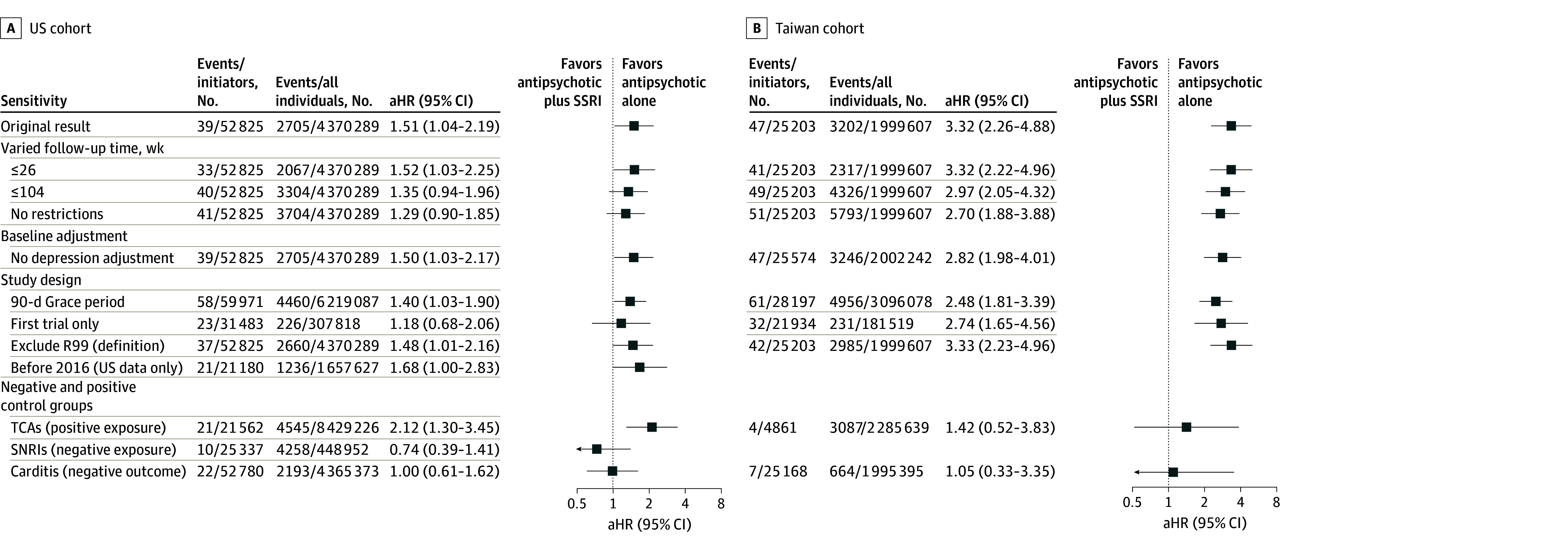
Per-Protocol Analysis of the Association Between Concomitant SSRI Use and VA or Sudden Death in Patients Receiving Antipsychotics (Sensitivity Analyses) Sensitivity analyses assessed the robustness of the primary 52-week per-protocol result under alternative specifications. Baseline depression was excluded from the IPW model, as depression may act as a mediator between SSRI use and VA or sudden death. In excluding R99, deaths coded as *International Statistical Classification of Diseases and Related Health Problems, Tenth Revision* code R99 (ill-defined or unknown cause) were removed from the composite VA or sudden death endpoint. Because US mortality data were available only through 2016, patients were censored at that date for before 2016. To probe residual uncontrolled confounding, TCAs replaced SSRIs as a positive exposure control, SNRIs replaced SSRIs as a negative exposure control, and incident carditis was analyzed as a negative outcome control. All adjusted HRs were estimated with IPW incorporating baseline covariates and time-varying factors relevant to treatment deviations and censoring; the Taiwan cohort additionally excluded observations with propensity scores outside the 2.5th to 97.5th percentiles. HR indicates hazard ratio; IPW, inverse probability weighting; SNRI, serotonin-norepinephrine reuptake inhibitor; SSRI, selective serotonin reuptake inhibitor; TCA, tricyclic antidepressant; VA, ventricular arrhythmia.

In subgroup analyses, patients under age 65 years in both cohorts were more susceptible to VA or sudden death with concomitant antipsychotic and SSRI use. However, sex-stratified estimates differed between cohorts (Figure 2; eFigure 3 in Supplement 1). In the sensitivity analyses, longer follow-up yielded smaller effect sizes in both cohorts. Results were similar across analyses omitting depression adjustment, alternating grace periods, excluding R99-coded outcomes, and restricting to pre-2016 US data (Figure 3; eFigure 4 and eTables 3 through 5 in Supplement 1).

In the control analyses, concurrent TCA use was associated with a higher VA or sudden death risk in the US (HR, 2.12; 95% CI, 1.30-3.45). In Taiwan, the estimate was imprecise with a wide 95% CI (HR, 1.42; 95% CI, 0.52-3.83). For SNRIs, the US cohort showed a lower but imprecise risk estimate (HR, 0.74; 95% CI, 0.39-1.41), while event numbers in Taiwan were insufficient to generate reliable estimates. Negative outcome control using carditis showed no association with concurrent SSRI use in either cohort (US: HR, 1.00; 95% CI, 0.61-1.62; Taiwan: HR, 1.05; 95% CI, 0.33-3.35).

## Discussion

This study found that SSRI initiation during antipsychotic treatment was associated with increased VA or sudden death risk, most pronounced for citalopram and escitalopram in the US cohort and for SSRIs overall in the Taiwan cohort. In the Taiwan cohort, conditional-risk SSRIs were associated with a higher VA or sudden death risk, which appeared to be driven largely by sertraline (HR, 4.16; 95% CI, 2.47-7.00); the finding, although consistent with a prior case report,^30^ requires further confirmation.

There were some notable differences between the 2 cohorts. For example, depression was more commonly recorded in Taiwan than in the US, particularly among SSRI initiators, which may reflect cross-system variations in clinical or coding practices. In Taiwan, reimbursement rules generally require prescriptions to align with approved indications, which may encourage more complete documentation of diagnosis in claims. The magnitude of increased risk also differed between cohorts. Differences in patient characteristics, baseline cardiometabolic risk, concomitant antipsychotic regimens, and comedications might have contributed to this heterogeneity.

Despite the elevated relative risk, absolute incidence proportion remained low (0.1% among SSRI initiators in the US and 0.2% in Taiwan). This pattern contrasts with epidemiologic data showing higher sudden death rates in Western than Asian populations (approximately 60 to 83 per 100 000 person-years in the US vs approximately 40 per 100 000 in Asian populations).^31,32^ This discrepancy should be interpreted cautiously because our estimates were incidence proportions rather than rates, relied on *ICD* codes whose accuracy may differ across health care systems, and were derived from antipsychotic users with distinct baseline risks.

Our findings align with clinical alerts regarding QT-prolonging risks of SSRIs, especially citalopram and escitalopram, but differ from prior studies reporting no increased arrhythmia risk with SSRI monotherapy. A previous Taiwanese study found no elevated risk of VA with overall SSRI use or with citalopram or escitalopram compared with other antidepressants.^33^ Similarly, studies from Denmark and the UK reported null findings, including at high doses.^10,34^ A likely explanation is the exposure contrast: we examined concurrent use with antipsychotics, where pharmacodynamic potentiation (eg, dual delayed rectifier potassium current inhibition) and pharmacokinetic interactions (eg, CYP2D6-mediated increases in antipsychotic concentrations) may increase risk compared with monotherapy settings (eTables 6 and 7 in Supplement 1).^2,7,16,35^

Subgroup analyses showed that risk elevation with concurrent SSRI and antipsychotic use was more pronounced in younger patients in both cohorts, whereas sex-stratified estimates differed between cohorts. Although baseline sudden death rates are typically higher in older adults and male patients,^36^ drug-induced, QT-mediated arrhythmias are more frequently documented in female patients, who tend to have longer baseline QT intervals and greater susceptibility to delayed rectifier potassium current blockade.^37,38^ At present, no established mechanism accounts for the higher risk observed in younger patients, underscoring the need for further study.

To our knowledge, this is the first study to apply sequential target trial emulation to evaluate the effect of adding a precipitant drug vs not adding it to ongoing treatment. Conventional designs are less suitable for addressing our study objective. An active-comparator new-user design (eg, SSRI vs SNRI initiation among antipsychotic users) may be underpowered due to the limited number of SNRI users available as comparators. Self-controlled case series design requires that the outcome not preclude subsequent exposure^39^; this assumption is violated for sudden death. Evaluating VA alone is also challenging because events are too rare for reliable estimates. Without an appropriate comparator, comparisons with nonusers can introduce immortal-time bias if not carefully addressed.^40^ Sequential target trial emulation helped mitigate these issues. By focusing on patients already receiving antipsychotics and defining follow-up from SSRI initiation, we isolated the incremental VA or sudden death risk associated with adding an SSRI, rather than having it obscured by the known baseline risk of antipsychotics. However, because we compare SSRI initiation vs no initiation among antipsychotic users, our estimand is conditional on antipsychotic use. Therefore, without SSRI-only or neither-drug groups, we could not quantify interaction on additive or multiplicative scales.^41^

### Strengths and Limitations

Our study has several strengths. First, it is the first observational study to quantify VA or sudden death risk with concomitant antipsychotic-SSRI therapy, addressing a key evidence gap for a common yet rarely evaluated regimen. Second, large US and Taiwan datasets enabled assessment of rare outcomes and supported generalizability across populations and health care systems. Taiwan’s inpatient medication records also reduce exposure misclassification and further strengthen robustness. Third, we used sequential target trial emulation to mitigate immortal time bias by aligning eligibility, treatment assignment, and follow-up start at each weekly decision point. This avoided biases that could arise when SSRI initiation was defined using posteligibility information, which might select individuals who remained event-free until initiation or misclassify event-free preinitiation time as exposed time.^40^ Finally, we conducted extensive sensitivity analyses and negative or positive control analyses to strengthen the validity of our results and provide greater confidence in the observed associations.

Several limitations should be acknowledged. First, event counts were modest despite large cohorts, limiting precision and preventing analyses separating VA from sudden death or estimating effects of individual SSRIs. Second, as an observational study, unmeasured and residual confounding remained possible despite extensive adjustment and control analyses. Our approach relies on correctly specified models, and estimates could be biased under misspecification. In the US cohort, socioeconomic status and race and/or ethnicity were unavailable and might confound SSRI initiation and VA or sudden death risk (eg, through differential access to care and monitoring). Generalizability may be limited because MarketScan includes commercially insured and Medicare supplemental beneficiaries. In contrast, NHIRD covers nearly the entire Taiwan population, supporting generalizability within Taiwan, although extrapolation to other health care systems may be limited. Third, our reference group comprised nonusers rather than users of an active comparator, raising concern about confounding by indication. We attempted to mitigate this by adjusting for baseline depression diagnosis and conducting subgroup analyses stratified by depression status. Fourth, genetic data such as CYP2D6 polymorphisms were unavailable, limiting our ability to assess potential population differences in susceptibility to the increased risk from concomitant drug use. Fifth, HRs can be difficult to interpret as a single measure because they are conditional on remaining event-free and may vary over time.^42^ Finally, we did not assess antipsychotic or SSRI dose, as dose titration is common in clinical practice and may not be accurately captured in our claims data.

## Conclusions

In this cohort study using a sequential target trial emulation framework, SSRI initiation during antipsychotic treatment was associated with increased VA or sudden death risk, particularly for citalopram and escitalopram in the US cohort and for all SSRIs in the Taiwan cohort. However, the absolute incidence of these events remained low. Clinicians may consider baseline risk assessment and close monitoring, particularly during early treatment and dose changes. When combination therapy is necessary, minimizing additional QT-prolonging medications and correcting modifiable risk factors may help mitigate risk. These findings also underscore the need for further research to guide safer, individualized treatment.

## Notes

Supplement 1. eFigure 1. Study Design Diagram of Initial Weekly Target Trial eFigure 2. Patient Selection and Person-Trial Flow in the US and Taiwan Cohorts eFigure 3. Intention-to-Treat Analysis of the Association Between Concomitant SSRI Use and VA/SD Among Patients on Antipsychotics: Subgroup Analyses eFigure 4. Intention-to-Treat Analysis of the Association Between Concomitant SSRI Use and VA/SD Among Patients on Antipsychotics: Sensitivity Analyses eTable 1. Summary of the Target Trial Protocol and Emulated Protocol eTable 2. Complete Baseline Characteristics of SSRI Initiators and Noninitiators at the Start of Trial Follow-Up eTable 3. Baseline Characteristics of SSRI Initiators and Noninitiators at the Start of Trial Follow-Up eTable 4. Baseline Characteristics of SNRI Initiators and Noninitiators at the Start of Trial Follow-Up in the US Cohort eTable 5. Baseline Characteristics of TCA Initiators and Noninitiators at the Start of Trial Follow-Up eTable 6. Study Antipsychotics: TdP Risk Classification, CYP450 Substrates, and Availability in Taiwan NHIRD and the US MarketScan Databases eTable 7. Study Antidepressants: Therapeutic Class, TdP Risk Classification, CYP450 Inhibition Profile, and Availability in Taiwan NHIRD and the US MarketScan Databases

Supplement 2. Data Sharing Statement
