# Supplementary material for: Ventricular Arrhythmia and Sudden Death Risk With Concomitant Antipsychotic and SSRI Use
Source: JAMA Netw Open. 2026 Apr 9;9(4):e266028. doi: 10.1001/jamanetworkopen.2026.6028 (PMC13067012; doi:10.1001/jamanetworkopen.2026.6028)
Supplement: Supplement 1. — eFigure 1. Study Design Diagram of Initial Weekly Target Trial eFigure 2. Patient Selection and Person-Trial Flow in the US and Taiwan Cohorts eFigure 3. Intention-to-Treat Analysis of the Association Between Concomitant SSRI Use and VA/SD Among Patients on Antipsychotics: Subgroup Analyses eFigure 4. Intention-to-Treat Analysis of the Association Between Concomitant SSRI Use and VA/SD Among Patients on Antipsychotics: Sensitivity Analyses eTable 1. Summary of the Target Trial Protocol and Emulated Protocol eTable 2. Complete Baseline Characteristics of SSRI Initiators and Noninitiators at the Start of Trial Follow-Up eTable 3. Baseline Characteristics of SSRI Initiators and Noninitiators at the Start of Trial Follow-Up eTable 4. Baseline Characteristics of SNRI Initiators and Noninitiators at the Start of Trial Follow-Up in the US Cohort eTable 5. Baseline Characteristics of TCA Initiators and Noninitiators at the Start of Trial Follow-Up eTable 6. Study Antipsychotics: TdP Risk Classification, CYP450 Substrates, and Availability in Taiwan NHIRD and the US MarketScan Databases eTable 7. Study Antidepressants: Therapeutic Class, TdP Risk Classification, CYP450 Inhibition Profile, and Availability in Taiwan NHIRD and the US MarketScan Databases [file jamanetwopen-e266028-s001.pdf]

Supplemental Online Content

Chien H, Lin S, Kung T, et al. Ventricular arrhythmia and sudden death risk with concomitant antipsychotic and SSRI use. *JAMA Netw Open.* 2026;9(4):e266028. doi:10.1001/jamanetworkopen.2026.6028

eFigure 1. Study Design Diagram of Initial Weekly Target Trial

eFigure 2. Patient Selection and Person-Trial Flow in the US and Taiwan Cohorts

eFigure 3. Intention-to-Treat Analysis of the Association Between Concomitant SSRI Use and VA/SD Among Patients on Antipsychotics: Subgroup Analyses

eFigure 4. Intention-to-Treat Analysis of the Association Between Concomitant SSRI Use and VA/SD Among Patients on Antipsychotics: Sensitivity Analyses

eTable 1. Summary of the Target Trial Protocol and Emulated Protocol

eTable 2. Complete Baseline Characteristics of SSRI Initiators and Noninitiators at the Start of Trial Follow-Up

eTable 3. Baseline Characteristics of SSRI Initiators and Noninitiators at the Start of Trial Follow-Up

eTable 4. Baseline Characteristics of SNRI Initiators and Noninitiators at the Start of Trial Follow-Up in the US Cohort

eTable 5. Baseline Characteristics of TCA Initiators and Noninitiators at the Start of Trial Follow-Up

eTable 6. Study Antipsychotics: TdP Risk Classification, CYP450 Substrates, and Availability in Taiwan NHIRD and the US MarketScan Databases

eTable 7. Study Antidepressants: Therapeutic Class, TdP Risk Classification, CYP450 Inhibition Profile, and Availability in Taiwan NHIRD and the US MarketScan Databases

This supplemental material has been provided by the authors to give readers additional information about their work.

© 2026 Chien H et al. *JAMA Network Open*.

eTable 1. Summary of the target trial protocol and emulated protocol

Component	Hypothetical target trial	Target trial emulation with observational data
**Eligibility criteria**	• Individuals aged 18 years or older with psychotic disorders, initiating antipsychotic treatment in an outpatient setting<br>• No antipsychotic or SSRI prescriptions in the 365 days before the cohort entry date, no history of VA/SD, and no concurrent use of multiple antipsychotics on the cohort entry date	Same as the target trial, with the following additional requirements:<br>• US cohort: ≥1 year of continuous enrollment in the health plan before the cohort entry date<br>• Taiwan cohort: Availability of sex information and exclusion of PRN antipsychotic use
**Treatment strategies**	Individuals receiving antipsychotics are randomly assigned to:<br>• Treatment group: SSRIs<br>• Control group: No SSRIs	Individuals receiving antipsychotics are classified into:<br>• Initiators: Started SSRI at Trial 1 (or subsequent person-trial)<br>• Non-initiator: Did not initiate SSRI at Trial 1 (or subsequent person-trial)
**Treatment assignment**	Randomly assigned to a strategy at baseline; individuals will be aware of their assigned strategy	Emulated using IPTW based on baseline covariates for each person-trial
**Follow-up**	Begins at randomization (time 0) and continues until the earliest occurrence of VA/SD, non-sudden death, discontinuation of the index antipsychotic, the end of the study period, or 52 weeks.	Same as the target trial, except time 0 is defined as the start of each person-trial week. Follow-up also ends upon health plan disenrollment in the US cohort
**Outcomes**	VA and SD	Same as the target trial
**Causal contrasts**	• Intention-to-treat effect: Effect of treatment assignment<br>• Per protocol effect: Effect of adhering to the assigned treatment	• Intention-to-treat effect<br>• Per protocol effect: Individuals are censored upon deviation from initial treatment in each person-trial; IPW is applied for censoring)
**Statistical analysis**	Cox proportional hazard model	Hazard ratio approximated via odds ratio from a pooled logistic regression model with a robust variance estimator to account for repeated measures and provide conservative 95% confidence intervals

Abbreviations: IPW, inverse probability weighting; IPTW, inverse probability of treatment weighting; PRN, pro re nata (as needed); SD, sudden death; SSRI, selective serotonin reuptake inhibitor; VA, ventricular arrhythmia.

© 2026 Chien H et al. *JAMA Network Open*.

(A)

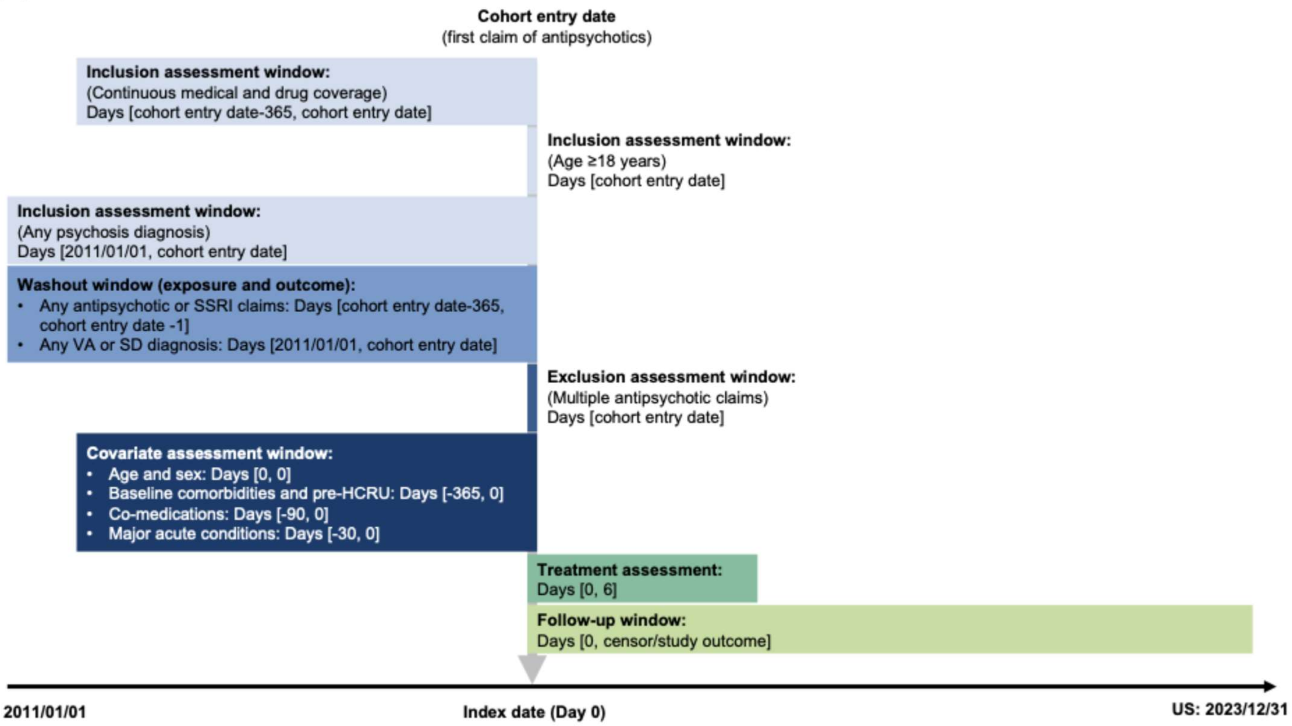

(B)

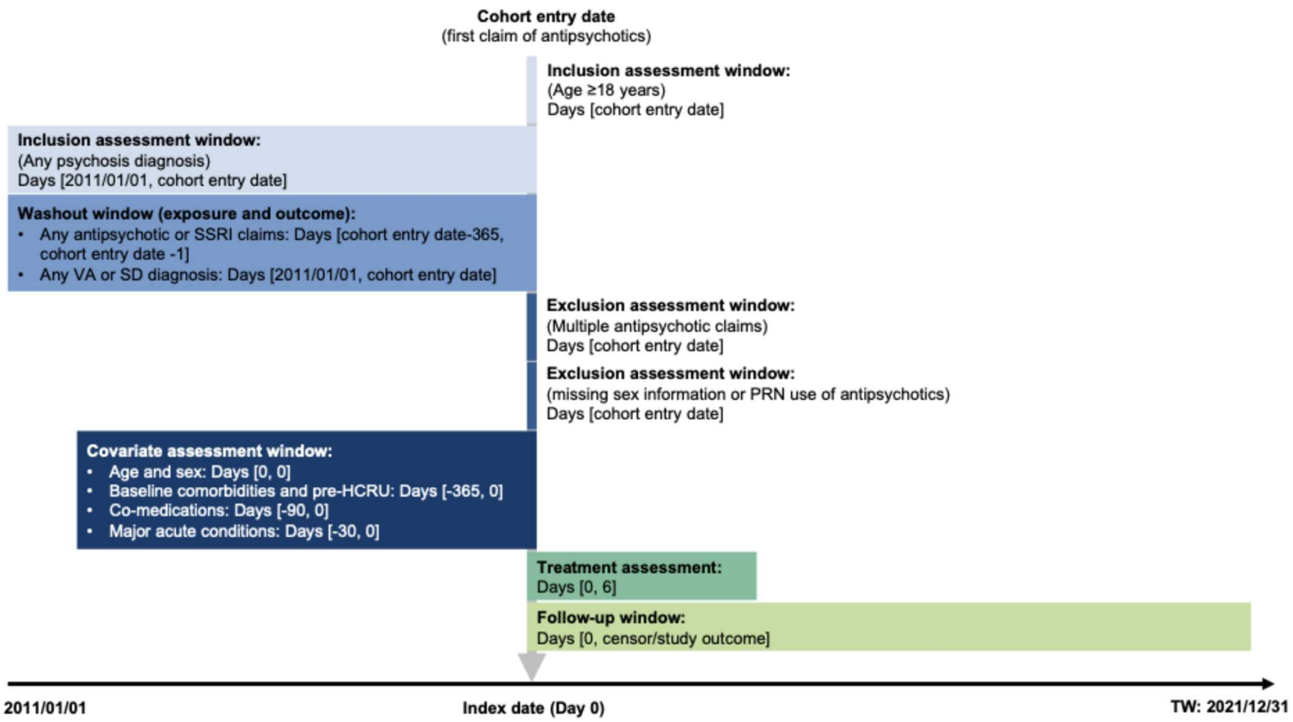

eFigure 1. Study design diagram of initial weekly target trial: (A) US cohort, (B) Taiwan cohort

The cohort entry date is defined as the first claim for an outpatient antipsychotic prescription. In the first weekly trial (Week 1), the index date (Day 0) coincides with the cohort entry date. In subsequent weekly trials, the trial-specific index date shifts forward by 7 days from the previous trial (e.g., Trial 2 = cohort entry + 7 days, Trial 3 = cohort entry + 14 days, and

© 2026 Chien H et al. *JAMA Network Open*.

so on). In each trial, patients who initiate SSRI treatment during the 7-day treatment assessment window (Days 0–6 from the index date) are classified as SSRI initiators, while those without SSRI initiation during this period are classified as non-initiators. If both SSRI initiation and the study outcome occur within the same week, classification is based on whichever event occurs first.

Abbreviations: HCRU, healthcare resource utilization; PRN, pro re nata (as needed); SD, sudden death; SSRI, selective serotonin reuptake inhibitor; TW, Taiwan; VA, ventricular arrhythmia.

© 2026 Chien H et al. *JAMA Network Open*.

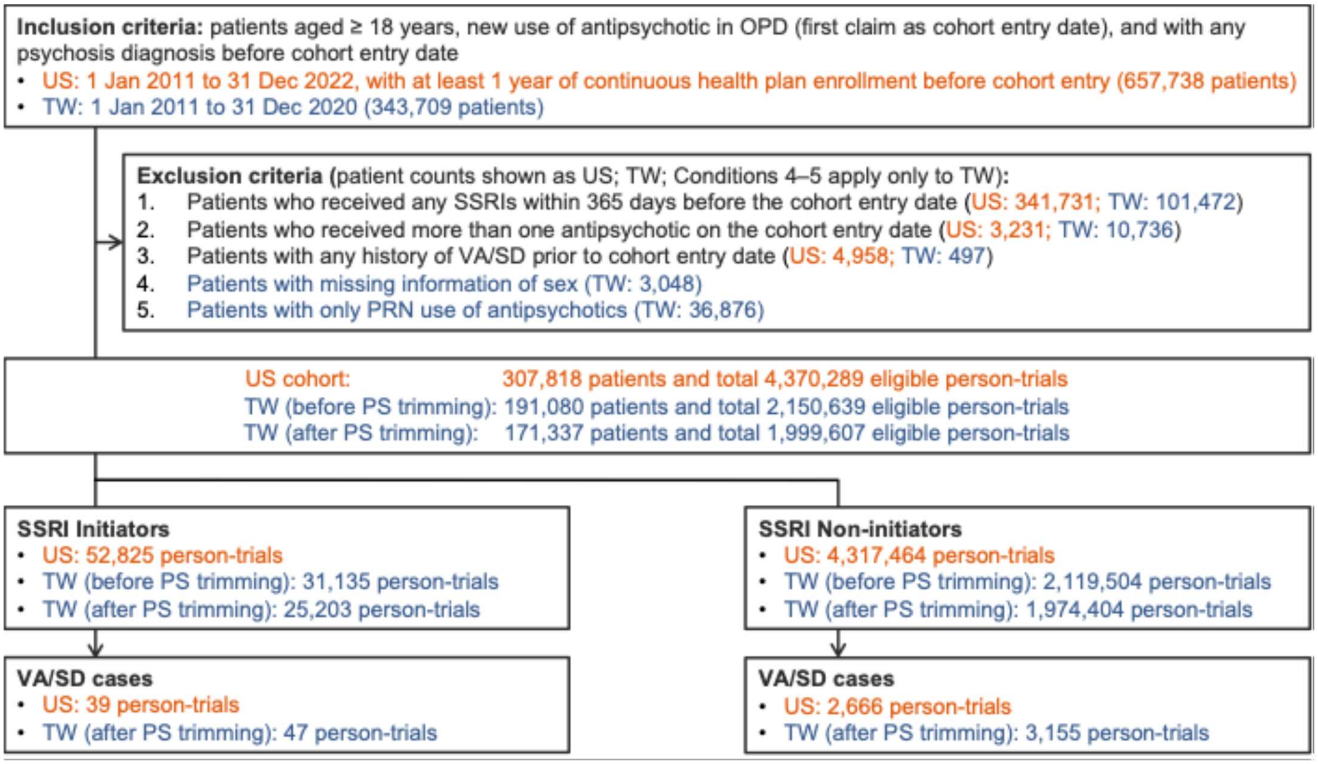

eFigure 2. Patient selection and person-trial flow in the US and Taiwan cohorts

Person trials were created by allowing reentry into weekly trials for patients continuing antipsychotic therapy.

Abbreviations: OPD, outpatient department; PRN, pro re nata; PS, propensity score; SSRI, selective serotonin reuptake inhibitor; TW, Taiwan; US, United States; VA/SD, ventricular arrhythmia or sudden death.

© 2026 Chien H et al. *JAMA Network Open*.

Subgroup	US cohort Events/ Initiators	US cohort Events/ All individuals	US cohort Adjusted HR (95% CI)	Taiwan cohort Events/ Initiators	Taiwan cohort Events/ All individuals	Taiwan cohort Adjusted HR (95% CI)
**Overall cohort**	**39/52,825**	**2,922/4,370,289**	**1.28 (0.91–1.81)**	**55/25,203**	**3,427/1,999,607**	**2.00 (1.47–2.72)**
**Sex**						
Male	19/23,143	1,801/1,696,109	0.88 (0.54–1.43)	34/10,139	1,836/896,011	2.62 (1.77–3.89)
Female	20/29,682	1,121/2,674,180	1.89 (1.15–3.11)	21/15,010	1,601/1,103,700	1.48 (0.90–2.42)
**Age**						
> 65 years	19/5,488	1,860/629,554	1.22 (0.74–2.02)	29/4,372	2,129/481,974	2.24 (1.47–3.40)
≤ 65 years	20/47,337	1,062/3,740,735	1.75 (1.09–2.82)	25/21,180	1,183/1,515,613	2.40 (1.52–3.80)
**CYP2D6 substrates**						
Yes	11/23,644	1,086/2,140,330	1.11 (0.59–2.11)	11/4,509	703/430,589	2.40 (1.22–4.73)
No	28/29,181	1,836/2,229,959	1.29 (0.85–1.95)	44/20,687	2,627/1,563,352	1.97 (1.40–2.79)
**Known risk**						
Yes	0/850	36/75,937		11/9,929	439/472,669	2.62 (1.06–6.45)
No	39/51,975	2,886/4,294,352	1.30 (0.91–1.84)	46/15,894	2,981/1,522,780	2.31 (1.65–3.23)
**Depression history**						
Yes	2/10,989	95/644,642	1.42 (0.32–6.29)	39/21,217	1,825/1,021,806	1.68 (1.14–2.47)
No	37/41,836	2,827/3,725,647	1.34 (0.94–1.91)	16/5,421	1,681/977,028	2.92 (1.71–4.97)

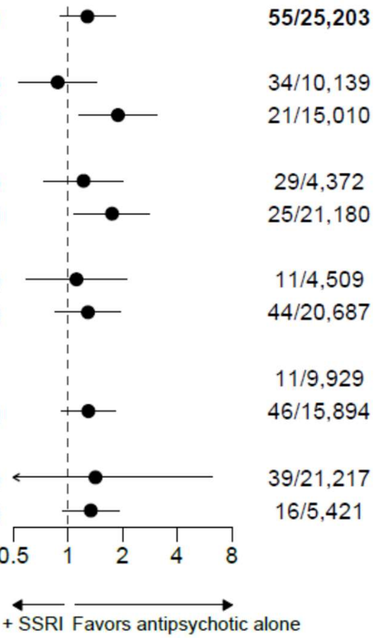

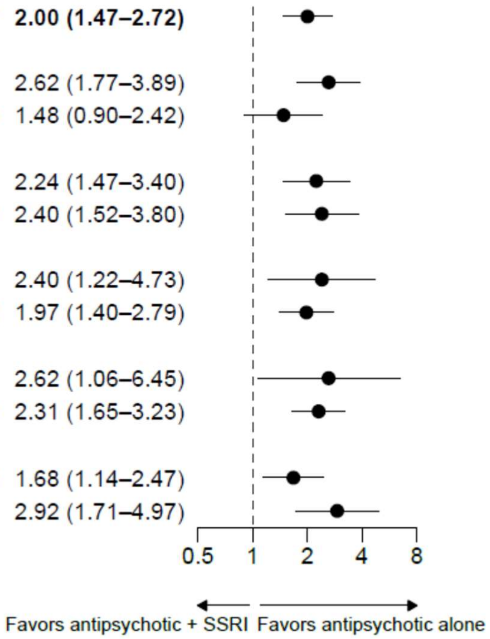

eFigure 3. Intention-to-treat analysis of the association between concomitant SSRI use and VA/SD among patients on antipsychotics: subgroup analyses

Age was defined as the age at the time of cohort entry (antipsychotic initiation). An antipsychotic was classified as a CYP2D6 substrate if it is primarily metabolized by the cytochrome P450 2D6 enzyme. Known risk refers to antipsychotics categorized by *CredibleMeds* as having a known risk of torsades de pointes. Depression history was defined as at least two outpatient diagnoses or one inpatient diagnosis within 365 days prior to the date of antipsychotic initiation. Adjusted HRs for the intention-to-treat analysis were estimated using inverse probability of treatment weighting based on baseline covariates. In the Taiwan cohort, patients with propensity scores outside the 2.5th and 97.5th percentiles were excluded to reduce potential confounding.

Abbreviations: CI, confidence interval; CYP2D6, cytochrome P450 2D6; HR, hazard ratio; SD, sudden death; SSRI, selective serotonin reuptake inhibitor; VA, ventricular arrhythmia.

© 2026 Chien H et al. *JAMA Network Open*.

Sensitivity	US cohort Events/ Initiators	US cohort Events/ All individuals	US cohort Adjusted HR (95% CI)	Taiwan cohort Events/ Initiators	Taiwan cohort Events/ All individuals	Taiwan cohort Adjusted HR (95% CI)
**Original result**	**39/52,825**	**2,922/4,370,289**	**1.28 (0.91–1.81)**	**55/25,203**	**3,427/1,999,607**	**2.00 (1.47–2.72)**
**Varied follow-up time**						
Up to 26 weeks	33/52,825	2,187/4,370,289	1.31 (0.91–1.89)	45/25,203	2,440/1,999,607	1.96 (1.42–2.72)
Up to 104 weeks	40/52,825	3,539/4,370,289	1.17 (0.83–1.66)	60/25,203	4,715/1,999,607	1.81 (1.35–2.42)
No restrictions	41/52,825	3,967/4,370,289	1.13 (0.80–1.59)	64/25,203	6,362/1,999,607	1.65 (1.25–2.19)
**Baseline adjustment**						
No depression adjustment	39/52,825	2,922/4,370,289	1.28 (0.91–1.81)	55/25,574	3,471/2,002,242	1.96 (1.44–2.66)
**Study Design**						
90-Day grace period	59/59,971	4,835/6,219,087	1.28 (0.96–1.69)	72/28,197	5,373/3,096,078	1.73 (1.33–2.24)
First trial only	23/31,483	240/307,818	1.09 (0.69–1.73)	37/21,934	253/181,519	2.05 (1.34–3.13)
Exclude R99 (definition)	37/52,825	2,874/4,370,289	1.24 (0.87–1.76)	48/25,203	3,204/1,999,607	1.87 (1.35–2.59)
Pre-2016 (US data only)	21/21,180	1,305/1,657,627	1.41 (0.85–2.34)			
**Negative and Positive Controls**						
TCAs (Positive exposure)	21/21,562	4,640/8,429,226	2.35 (1.51–3.66)	5/4,861	3,135/2,285,639	1.20 (0.49–2.89)
SNRIs (Negative exposure)	11/25,337	4,302/448,952	0.81 (0.44–1.49)			
Carditis (Negative outcome)	22/52,780	2,297/4,365,373	0.91 (0.58–1.44)	8/25,168	668/1,995,395	0.86 (0.30–2.47)

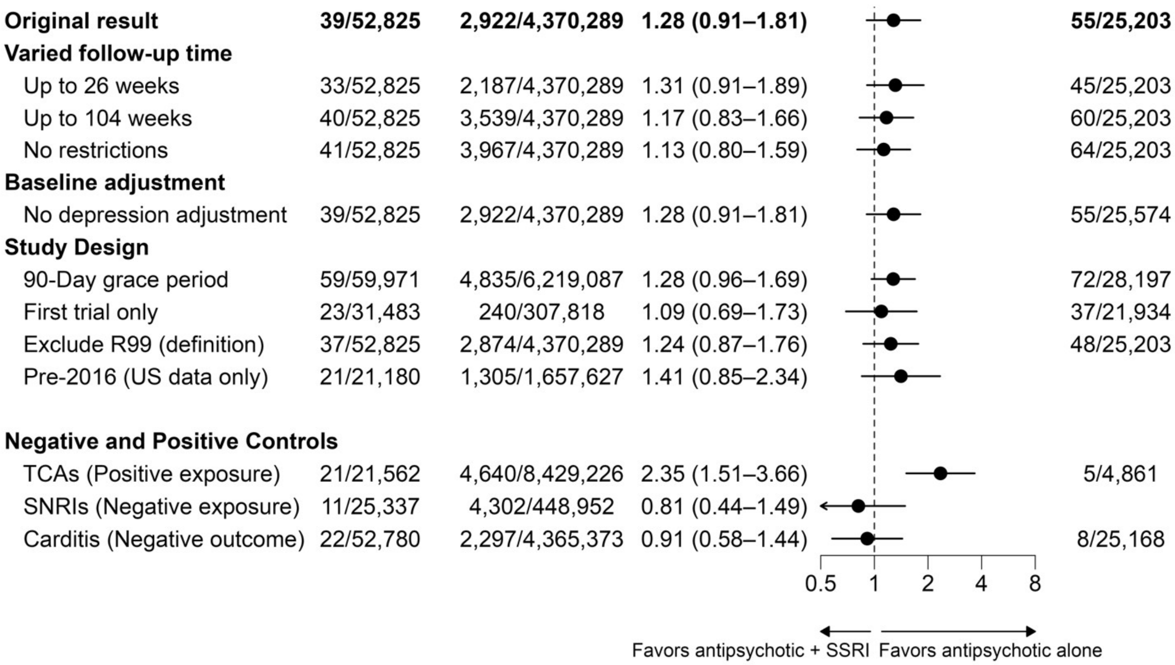

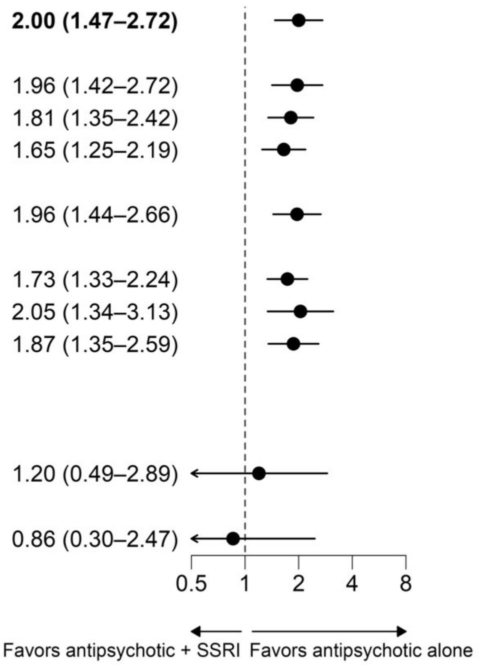

eFigure 4. Intention-to-treat analysis of the association between concomitant SSRI use and VA/SD among patients on antipsychotics: sensitivity analyses

Sensitivity analyses assessed the robustness of the primary 52-week per-protocol result under alternative specifications. (i) **Varied follow-up:** the risk window was shortened to ≤ 26 weeks, extended to ≤ 104 weeks, and left unrestricted (follow-up continued until outcome, treatment discontinuation, death, or study end). (ii) **No depression adjustment:** baseline depression was excluded from the IPW model, as depression may act as a mediator between SSRI use and VA/SD (iii) **90-day grace period:** the allowable gap between medication dispensing that defined continuous exposure was widened from 30 days to 90 days. (iv) **First-trial only:** analyses were limited to the first treatment episode for each patient. (v) **Exclude R99:** deaths coded as ICD-10-CM R99 (ill-defined/unknown cause) were removed from the composite VA/SD end-point. (vi) **Pre-2016 (US data only):** because US mortality data were available only through 2016, patients were censored at that date. (vii) **Negative and positive controls:** to probe residual uncontrolled confounding, TCAs replaced SSRIs as a positive exposure control, SNRIs replaced SSRIs as a negative exposure control, and incident carditis was analyzed as a negative outcome control. All adjusted HRs were

© 2026 Chien H et al. *JAMA Network Open*.

estimated with IPW incorporating baseline covariates and time-varying factors relevant to treatment deviations and censoring; the Taiwan cohort additionally excluded observations with propensity scores outside the 2.5th–97.5th percentiles.

Abbreviations: CI, confidence interval; HR, hazard ratio; IPW, inverse probability weighting; SD, sudden death; SNRI, serotonin-norepinephrine reuptake inhibitor; SSRI, selective serotonin reuptake inhibitor; TCA, tricyclic antidepressant; VA, ventricular arrhythmia.

© 2026 Chien H et al. *JAMA Network Open*.

eTable 2. Complete baseline characteristics of SSRI initiators and non-initiators at the start of trial follow-up: (A) US cohort, (B) Taiwan cohort (main analysis)

(A) US cohort

Covariates	Before weighting			After weighting		
	Initiators (n=52,825)	Non-initiators (n=4,317,464)	SMD	Initiators (n=53,699)	Non-initiators (n=4,317,456)	SMD
**Age, mean ± SD**	41.0 ± 18.6	46.7 ± 18.8	-0.31	46.3 ± 19.0	46.7 ± 18.8	-0.02
**Sex, n (%)**			-0.10			-0.02
Female	29,682 (56.2)	2,644,498 (61.3)		32,262 (60.1)	2,641,838 (61.2)	
Male	23,143 (43.8)	1,672,966 (38.7)		21,437 (39.9)	1,675,618 (38.8)	
**Antipsychotics, n (%)**			0.36			0.06
Aripiprazole	13,426 (25.4)	1,405,465 (32.6)		16,422 (30.6)	1,401,727 (32.5)	
Brexpiprazole	456 (0.9)	77,777 (1.8)		996 (1.9)	77,287 (1.8)	
Cariprazine	470 (0.9)	79,504 (1.8)		1,144 (2.1)	79,008 (1.8)	
Chlorpromazine	138 (0.3)	14,907 (0.3)		176 (0.3)	14,863 (0.3)	
Clozapine	53 (0.1)	6,032 (0.1)		78 (0.1)	6,011 (0.1)	
Droperidol	0 (0.0)	3 (0.0)		0 (0.0)	3 (0.0)	
Fluphenazine	60 (0.1)	5,658 (0.1)		61 (0.1)	5,649 (0.1)	
Haloperidol	693 (1.3)	56,378 (1.3)		719 (1.3)	56,381 (1.3)	
Iloperidone	12 (0.0)	2,212 (0.1)		39 (0.1)	2,197 (0.1)	
Loxapine	45 (0.1)	3,562 (0.1)		43 (0.1)	3,563 (0.1)	
Lumateperone	5 (0.0)	1,598 (0.0)		16 (0.0)	1,584 (0.0)	
Lurasidone	1,041 (2.0)	172,104 (4.0)		2,490 (4.6)	171,053 (4.0)	
Olanzapine	6,823 (12.9)	319,874 (7.4)		4,332 (8.1)	322,762 (7.5)	
Paliperidone	186 (0.4)	19,874 (0.5)		256 (0.5)	19,818 (0.5)	
Perphenazine	174 (0.3)	15,777 (0.4)		198 (0.4)	15,758 (0.4)	
Pimavanserin	10 (0.0)	2,503 (0.1)		32 (0.1)	2,483 (0.1)	
Pimozide	10 (0.0)	1,988 (0.0)		30 (0.1)	1,974 (0.0)	
Prochlorperazine	1,157 (2.2)	217,879 (5.0)		2,947 (5.5)	216,389 (5.0)	
Quetiapine	18,808 (35.6)	1,361,673 (31.5)		16,667 (31.0)	1,363,783 (31.6)	
Risperidone	8,268 (15.7)	462,564 (10.7)		5,888 (11.0)	465,142 (10.8)	
Thioridazine	9 (0.0)	1,811 (0.0)		17 (0.0)	1,798 (0.0)	
Trifluoperazine	26 (0.0)	3,054 (0.1)		40 (0.1)	3,043 (0.1)	
Ziprasidone	955 (1.8)	85,267 (2.0)		1,107 (2.1)	85,180 (2.0)	
**Clinical variables**						
CCI score, n (%)			0.07			0.02
0	49,719 (94.1)	4,025,110 (93.2)		49,845 (92.8)	4,025,566 (93.2)	
1	2,271 (4.3)	187,232 (4.3)		2,386 (4.4)	187,213 (4.3)	
2	736 (1.4)	98,191 (2.3)		1,361 (2.5)	97,732 (2.3)	
≥ 3	99 (0.2)	6,931 (0.2)		106 (0.2)	6,945 (0.2)	
**Comorbidities in CCI, n (%)**						

© 2026 Chien H et al. *JAMA Network Open*.

Covariates	Before weighting			After weighting		
	Initiators (n=52,825)	Non-initiators (n=4,317,464)	SMD	Initiators (n=53,699)	Non-initiators (n=4,317,456)	SMD
Cancer	558 (1.1)	76,907 (1.8)	-0.06	1,117 (2.1)	76,517 (1.8)	0.02
Metastatic cancer	27 (0.1)	2,391 (0.1)	-0.00	50 (0.1)	2,385 (0.1)	0.01
Cerebral vascular disease	73 (0.1)	6,075 (0.1)	-0.00	77 (0.1)	6,070 (0.1)	0.00
Chronic pulmonary disease	159 (0.3)	11,868 (0.3)	0.00	146 (0.3)	11,877 (0.3)	-0.00
Congestive heart failure	27 (0.1)	3,623 (0.1)	-0.01	35 (0.1)	3,619 (0.1)	-0.01
Dementia	546 (1.0)	47,271 (1.1)	-0.01	569 (1.1)	47,307 (1.1)	-0.00
Diabetes without chronic complication	1,320 (2.5)	106,358 (2.5)	0.00	1,411 (2.6)	106,303 (2.5)	0.01
Diabetes with chronic complication	144 (0.3)	16,430 (0.4)	-0.02	194 (0.4)	16,373 (0.4)	-0.00
Hemiplegia or paraplegia	30 (0.1)	2,198 (0.1)	0.00	40 (0.1)	2,194 (0.1)	0.01
HIV/AIDS	66 (0.1)	4,240 (0.1)	0.01	50 (0.1)	4,260 (0.1)	-0.00
Mild liver disease	81 (0.2)	5,684 (0.1)	0.01	70 (0.1)	5,691 (0.1)	-0.00
Severe liver disease	3 (0.0)	190 (0.0)	0.00	3 (0.0)	191 (0.0)	0.00
Myocardial infarction	27 (0.1)	2,169 (0.1)	0.00	30 (0.1)	2,168 (0.1)	0.00
Peptic ulcer	19 (0.0)	1,237 (0.0)	0.00	19 (0.0)	1,237 (0.0)	0.00
Peripheral artery disease	18 (0.0)	1,957 (0.0)	-0.01	26 (0.0)	1,952 (0.0)	0.00
Renal disease	8 (0.0)	2,500 (0.1)	-0.02	13 (0.0)	2,493 (0.1)	-0.02
Rheumatic disease	13 (0.0)	1,964 (0.0)	-0.01	19 (0.0)	1,962 (0.0)	-0.01
**Cardiometabolic comorbidities, n (%)**						
Atrial fibrillation	40 (0.1)	3,674 (0.1)	-0.00	48 (0.1)	3,673 (0.1)	0.00
Coronary artery disease	71 (0.1)	6,975 (0.2)	-0.01	82 (0.2)	6,965 (0.2)	-0.00
Diabetes mellitus	1,567 (3.0)	134,145 (3.1)	-0.01	1,719 (3.2)	134,010 (3.1)	0.01
Endocarditis	2 (0.0)	273 (0.0)	-0.00	1 (0.0)	274 (0.0)	-0.01
ESRD	1 (0.0)	564 (0.0)	-0.01	1 (0.0)	562 (0.0)	-0.01
Hyperlipidemia	1,253 (2.4)	99,967 (2.3)	0.00	1,251 (2.3)	99,984 (2.3)	0.00
Hypertension	450 (0.9)	45,925 (1.1)	-0.02	496 (0.9)	45,872 (1.1)	-0.01
Stroke	17 (0.0)	1,422 (0.0)	-0.00	21 (0.0)	1,421 (0.0)	0.00
Valvular heart disease	33 (0.1)	3,750 (0.1)	-0.01	39 (0.1)	3,749 (0.1)	-0.01
**Comorbidities associated with TdP, n (%)**						
AV block	0 (0.0)	52 (0.0)	-0.00	0 (0.0)	52 (0.0)	-0.00
Ankylosing spondylitis	0 (0.0)	63 (0.0)	-0.01	0 (0.0)	63 (0.0)	-0.01
Hyperparathyroidism	13 (0.0)	621 (0.0)	0.01	13 (0.0)	621 (0.0)	0.01
Hypothyroidism	1,113 (2.1)	93,506 (2.2)	-0.00	1,161 (2.2)	93,477 (2.2)	-0.00
Panhypopituitarism	5 (0.0)	197 (0.0)	0.01	4 (0.0)	197 (0.0)	0.00
Rheumatic arthritis	2 (0.0)	807 (0.0)	-0.01	4 (0.0)	805 (0.0)	-0.01
**Psychiatric comorbidities, n (%)**						
Alcohol use disorder	2,092 (4.0)	91,953 (2.1)	0.11	1,156 (2.2)	92,910 (2.2)	0.00

© 2026 Chien H et al. *JAMA Network Open*.

Covariates	Before weighting			After weighting		
	Initiators (n=52,825)	Non-initiators (n=4,317,464)	SMD	Initiators (n=53,699)	Non-initiators (n=4,317,456)	SMD
Anxiety	867 (1.6)	57,178 (1.3)	0.03	653 (1.2)	57,341 (1.3)	-0.01
Depression	11,316 (21.4)	655,990 (15.2)	0.16	7,036 (13.1)	659,199 (15.3)	-0.06
Schizophrenia	1,031 (2.0)	70,492 (1.6)	0.02	960 (1.8)	70,660 (1.6)	0.01
**Co-medications, n (%)**						
Medication with known risk of QT-prolongation	6,399 (12.1)	434,188 (10.1)	0.07	5,423 (10.1)	435,262 (10.1)	0.00
Medications as strong CYP inhibitors						
CYP1A2 inhibitors	801 (1.5)	50,501 (1.2)	0.03	610 (1.1)	50,681 (1.2)	-0.00
CYP2D6 inhibitors	3,573 (6.8)	441,299 (10.2)	-0.12	6,043 (11.3)	439,498 (10.2)	0.03
CYP3A4 inhibitors	166 (0.3)	12,212 (0.3)	0.01	161 (0.3)	12,228 (0.3)	0.00
Other antidepressants	12,296 (23.3)	1,547,382 (35.8)	-0.28	18,909 (35.2)	1,540,819 (35.7)	-0.01
**HCRU in prior 1 year, mean ± SD**						
OPD visits	15.9 ± 17.3	21.4 ± 19.6	-0.30	21.5 ± 20.3	21.3 ± 19.6	0.01
ED visits	1.1 ± 2.1	1.0 ± 2.0	0.07	1.1 ± 2.2	1.0 ± 2.0	0.04
Total hospitalization stays	4.9 ± 12.0	3.4 ± 9.4	0.14	3.9 ± 11.5	3.4 ± 9.4	0.04

(B) Taiwan cohort

Covariates	Before weighting			After weighting		
	Initiators (n=31,135)	Non-initiators (n=2,119,504)	SMD	Initiators (n=31,915)	Non-initiators (n=1,967,692)	SMD
**Age, mean ± SD**	45.6 ± 18.1	51.1 ± 18.3	-0.3	51.8 ± 18.8	51.1 ± 18.3	0.04
**Sex, n (%)**			0.09			0
Female	18,545 (59.6)	1,165,919 (55.0)		17,409 (54.5)	1,073,587 (54.6)	
Male	12,590 (40.4)	953,585 (45.0)		14,506 (45.5)	894,105 (45.4)	
**Antipsychotics, n (%)**			0.56			0.06
Amisulpride	628 (2.0)	53,553 (2.5)		884 (2.8)	52,125 (2.6)	
Aripiprazole	2,814 (9.0)	176,698 (8.3)		2,628 (8.2)	170,994 (8.7)	
Flupentixol	5,122 (16.5)	200,258 (9.4)		3,031 (9.5)	188,172 (9.6)	
Haloperidol	237 (0.8)	30,490 (1.4)		490 (1.5)	29,015 (1.5)	
Olanzapine	1,209 (3.9)	79,540 (3.8)		1,165 (3.7)	76,117 (3.9)	
Prochlorperazine	312 (1.0)	144,300 (6.8)		1,628 (5.1)	101,115 (5.1)	
Quetiapine	6,477 (20.8)	670,446 (31.6)		11,262 (35.3)	646,247 (32.8)	
Risperidone	2,191 (7.0)	232,550 (11.0)		3,390 (10.6)	226,650 (11.5)	
Sulpiride	11,361 (36.5)	440,844 (20.8)		5,953 (18.7)	390,656 (19.9)	
Other antipsychotics*	784 (2.5)	90,825 (4.3)		1,484 (4.6)	86,601 (4.4)	
**Clinical variables**						
CCI score, n (%)			0.21			0.08

© 2026 Chien H et al. *JAMA Network Open*.

Covariates	Before weighting			After weighting		
	Initiators (n=31,135)	Non-initiators (n=2,119,504)	SMD	Initiators (n=31,915)	Non-initiators (n=1,967,692)	SMD
0	21,481 (69.0)	1,259,573 (59.4)		17,977 (56.3)	1,176,743 (59.8)	
1	4,894 (15.7)	397,139 (18.7)		6,234 (19.5)	368,000 (18.7)	
2	2,431 (7.8)	222,095 (10.5)		3,556 (11.1)	204,297 (10.4)	
≥ 3	2,329 (7.5)	240,697 (11.4)		4,148 (13.0)	218,652 (11.1)	
**Comorbidities in CCI, n (%)**						
Cancer	1,030 (3.3)	93,785 (4.4)	-0.06	1,593 (5.0)	83,907 (4.3)	0.03
Metastatic cancer	124 (0.4)	12,577 (0.6)	-0.03	187 (0.6)	10,177 (0.5)	0.01
Cerebral vascular disease	1,468 (4.7)	148,540 (7.0)	-0.1	2,701 (8.5)	136,212 (6.9)	0.06
Chronic pulmonary disease	1,731 (5.6)	158,296 (7.5)	-0.08	2,461 (7.7)	145,456 (7.4)	0.01
Congestive heart failure	583 (1.9)	53,767 (2.5)	-0.05	929 (2.9)	49,671 (2.5)	0.02
Dementia	805 (2.6)	141,828 (6.7)	-0.2	2,369 (7.4)	128,094 (6.5)	0.04
Diabetes without chronic complication	2,773 (8.9)	256,261 (12.1)	-0.1	4,201 (13.2)	235,327 (12.0)	0.04
Diabetes with chronic complication	858 (2.8)	81,544 (3.8)	-0.06	1,259 (3.9)	74,190 (3.8)	0.01
Hemiplegia or paraplegia	155 (0.5)	16,250 (0.8)	-0.03	250 (0.8)	14,953 (0.8)	0
HIV/AIDS	108 (0.3)	9,674 (0.5)	-0.02	135 (0.4)	9,304 (0.5)	-0.01
Mild liver disease	1,986 (6.4)	165,703 (7.8)	-0.06	2,751 (8.6)	153,587 (7.8)	0.03
Severe liver disease	52 (0.2)	4,722 (0.2)	-0.01	88 (0.3)	4,407 (0.2)	0.01
Myocardial infarction	131 (0.4)	10,451 (0.5)	-0.01	244 (0.8)	9,479 (0.5)	0.04
Peptic ulcer	2,647 (8.5)	233,757 (11.0)	-0.09	3,588 (11.2)	213,933 (10.9)	0.01
Peripheral artery disease	285 (0.9)	25,673 (1.2)	-0.03	449 (1.4)	23,440 (1.2)	0.02
Renal disease	795 (2.6)	76,178 (3.6)	-0.06	1,319 (4.1)	69,914 (3.6)	0.03
Rheumatic disease	641 (2.1)	49,845 (2.4)	-0.02	836 (2.6)	45,677 (2.3)	0.02
**Cardiometabolic comorbidities, n (%)**						
Atrial fibrillation	263 (0.8)	21,587 (1.0)	-0.02	372 (1.2)	19,807 (1.0)	0.02
Coronary artery disease	1,671 (5.4)	148,732 (7.0)	-0.07	2,545 (8.0)	136,708 (6.9)	0.04
Diabetes mellitus	3,010 (9.7)	280,233 (13.2)	-0.11	4,527 (14.2)	257,058 (13.1)	0.03
Endocarditis	27 (0.1)	2,888 (0.1)	-0.01	44 (0.1)	2,541 (0.1)	0
ESRD	218 (0.7)	20,879 (1.0)	-0.03	367 (1.2)	18,945 (1.0)	0.02
Hyperlipidemia	3,704 (11.9)	307,412 (14.5)	-0.08	5,166 (16.2)	284,286 (14.4)	0.05
Hypertension	5,959 (19.1)	550,327 (26.0)	-0.16	9,015 (28.2)	507,226 (25.8)	0.06
Stroke	585 (1.9)	59,485 (2.8)	-0.06	1,075 (3.4)	54,692 (2.8)	0.03
Valvular heart disease	319 (1.0)	26,437 (1.2)	-0.02	463 (1.5)	24,277 (1.2)	0.02
**Comorbidities associated with TdP, n (%)**						
AV block	17 (0.1)	642 (0.0)	0.01	25 (0.1)	591 (0.0)	0.02
Ankylosing spondylitis	62 (0.2)	5,674 (0.3)	-0.01	115 (0.4)	5,152 (0.3)	0.02
Hyperparathyroidism	25 (0.1)	1,540 (0.1)	0	41 (0.1)	1,476 (0.1)	0.02

© 2026 Chien H et al. *JAMA Network Open*.

Covariates	Before weighting			After weighting		
	Initiators (n=31,135)	Non-initiators (n=2,119,504)	SMD	Initiators (n=31,915)	Non-initiators (n=1,967,692)	SMD
Hypothyroidism	241 (0.8)	22,782 (1.1)	-0.03	321 (1.0)	20,947 (1.1)	-0.01
Panhypopituitarism	4 (0.0)	176 (0.0)	0	5 (0.0)	173 (0.0)	0.01
Rheumatic arthritis	186 (0.6)	15,373 (0.7)	-0.02	281 (0.9)	13,818 (0.7)	0.02
**Psychiatric comorbidities, n (%)**						
Alcohol use disorder	919 (3.0)	73,143 (3.5)	-0.03	1,332 (4.2)	68,661 (3.5)	0.04
Anxiety	13,233 (42.5)	736,443 (34.7)	0.16	12,234 (38.3)	693,762 (35.3)	0.06
Depression	24,840 (79.8)	1,082,804 (51.1)	0.63	16,542 (51.8)	1,002,993 (51.0)	0.02
Schizophrenia	1,610 (5.2)	285,856 (13.5)	-0.29	3,908 (12.2)	272,186 (13.8)	-0.05
**Co-medications, n (%)**						
Medication with known risk of QT-prolongation	4,779 (15.3)	334,292 (15.8)	-0.01	5,574 (17.5)	302,761 (15.4)	0.06
Medications as strong CYP inhibitors						
CYP1A2 inhibitors	238 (0.8)	18,444 (0.9)	-0.01	336 (1.1)	16,419 (0.8)	0.02
CYP2D6 inhibitors	1,336 (4.3)	101,621 (4.8)	-0.02	2,319 (7.3)	96,741 (4.9)	0.1
CYP3A4 inhibitors	349 (1.1)	28,218 (1.3)	-0.02	463 (1.5)	25,778 (1.3)	0.01
Other antidepressants	7,707 (24.8)	795,928 (37.6)	-0.28	13,301 (41.7)	739,098 (37.6)	0.08
**HCRU in prior 1 year, mean ± SD**						
OPD visits	17.1 ± 15.6	21.9 ± 17.0	-0.29	23.0 ± 18.0	21.7 ± 17.0	0.07
ED visits	0.6 ± 1.2	0.6 ± 1.5	-0.03	0.7 ± 1.5	0.6 ± 1.4	0.07
Total hospitalization stays	1.9 ± 10.6	3.0 ± 15.3	-0.09	3.3 ± 15.0	3.0 ± 15.3	0.02

n represents the number of person-trials; individuals could contribute to multiple non-initiator trials until SSRI initiation or censoring.

*'Other antipsychotics' category includes the following drugs: brexpiprazole, chlorpromazine, chlorprothixene, clozapine, clothiapine, droperidol, fluphenazine, loxapine, lurasidone, paliperidone, perphenazine, pimozide, thioridazine, trifluoperazine, ziprasidone, and zotepine. These drugs were grouped together due to their relatively low frequency of use in the study population.

Abbreviations: AV block, atrioventricular block; CCI, Charlson comorbidity index; CYP, cytochrome P450; ED, emergency department; ESRD, end-stage renal disease; HCRU, healthcare resource utilization; HIV/AIDS, human immunodeficiency virus/acquired immunodeficiency syndrome; OPD, outpatient department; SD, standard deviation; SMD, standardized mean difference; SSRI, selective serotonin reuptake inhibitor; TdP, torsades de pointes.

© 2026 Chien H et al. *JAMA Network Open*.

eTable 3. Baseline characteristics of SSRI initiators and non-initiators at the start of trial follow-up: (A) US cohort, (B) Taiwan cohort (sensitivity analysis with varied grace periods)

(A) US cohort

Covariates	Before weighting			After weighting		
	Initiators (n=59,971)	Non-initiators (n=6,159,116)	SMD	Initiators (n=60,695)	Non-initiators (n=6,159,110)	SMD
**Age, mean ± SD**	41.3 ± 18.5	46.4 ± 18.7	-0.28	46.0 ± 18.8	46.4 ± 18.7	-0.02
**Sex, n (%)**			-0.09			-0.02
Female	34,200 (57.0)	3,777,582 (61.3)		36,603 (60.3)	3,775,011 (61.3)	
Male	25,771 (43.0)	2,381,534 (38.7)		24,092 (39.7)	2,384,099 (38.7)	
**Antipsychotics, n (%)**			0.33			0.04
Aripiprazole	15,612 (26.0)	1,904,834 (30.9)		18,023 (29.7)	1,901,919 (30.9)	
Brexpiprazole	576 (1.0)	101,910 (1.7)		1,037 (1.7)	101,498 (1.6)	
Cariprazine	619 (1.0)	108,349 (1.8)		1,186 (2.0)	107,918 (1.8)	
Chlorpromazine	171 (0.3)	27,681 (0.4)		253 (0.4)	27,583 (0.4)	
Clozapine	55 (0.1)	7,015 (0.1)		70 (0.1)	7,002 (0.1)	
Droperidol	0 (0.0)	3 (0.0)		0 (0.0)	3 (0.0)	
Fluphenazine	65 (0.1)	8,253 (0.1)		68 (0.1)	8,238 (0.1)	
Haloperidol	780 (1.3)	84,514 (1.4)		856 (1.4)	84,472 (1.4)	
Iloperidone	16 (0.0)	3,221 (0.1)		40 (0.1)	3,206 (0.1)	
Loxapine	53 (0.1)	5,107 (0.1)		47 (0.1)	5,110 (0.1)	
Lumateperone	8 (0.0)	1,968 (0.0)		20 (0.0)	1,957 (0.0)	
Lurasidone	1,293 (2.2)	232,520 (3.8)		2,575 (4.2)	231,559 (3.8)	
Olanzapine	7,311 (12.2)	455,919 (7.4)		4,770 (7.9)	458,771 (7.4)	
Paliperidone	209 (0.3)	26,955 (0.4)		279 (0.5)	26,902 (0.4)	
Perphenazine	200 (0.3)	22,822 (0.4)		223 (0.4)	22,800 (0.4)	
Pimavanserin	13 (0.0)	3,016 (0.0)		31 (0.1)	3,000 (0.0)	
Pimozide	14 (0.0)	3,072 (0.0)		34 (0.1)	3,056 (0.0)	
Prochlorperazine	2,085 (3.5)	490,084 (8.0)		5,048 (8.3)	487,424 (7.9)	
Quetiapine	20,861 (34.8)	1,905,464 (30.9)		18,456 (30.4)	1,907,741 (31.0)	
Risperidone	8,915 (14.9)	641,232 (10.4)		6,408 (10.6)	643,878 (10.5)	
Thioridazine	12 (0.0)	2,617 (0.0)		24 (0.0)	2,604 (0.0)	
Trifluoperazine	31 (0.1)	4,369 (0.1)		46 (0.1)	4,358 (0.1)	
Ziprasidone	1,072 (1.8)	118,191 (1.9)		1,199 (2.0)	118,113 (1.9)	
**Clinical variables**						
CCI score, n (%)			0.08			0.01
0	56,322 (93.9)	5,706,791 (92.7)		56,080 (92.4)	5,707,531 (92.7)	
1	2,531 (4.2)	261,103 (4.2)		2,605 (4.3)	261,092 (4.2)	
2	1,004 (1.7)	180,455 (2.9)		1,885 (3.1)	179,710 (2.9)	
≥ 3	114 (0.2)	10,767 (0.2)		125 (0.2)	10,776 (0.2)	

© 2026 Chien H et al. *JAMA Network Open*.

Covariates	Before weighting			After weighting		
	Initiators (n=59,971)	Non-initiators (n=6,159,116)	SMD	Initiators (n=60,695)	Non-initiators (n=6,159,110)	SMD
**Comorbidities in CCI, n (%)**						
Cancer	805 (1.3)	150,181 (2.4)	-0.08	1,618 (2.7)	149,512 (2.4)	0.02
Metastatic cancer	35 (0.1)	4,050 (0.1)	-0.00	62 (0.1)	4,039 (0.1)	0.01
Cerebral vascular disease	83 (0.1)	8,350 (0.1)	0.00	86 (0.1)	8,345 (0.1)	0.00
Chronic pulmonary disease	179 (0.3)	18,316 (0.3)	0.00	167 (0.3)	18,321 (0.3)	-0.00
Congestive heart failure	30 (0.1)	5,299 (0.1)	-0.01	35 (0.1)	5,296 (0.1)	-0.01
Dementia	603 (1.0)	61,526 (1.0)	0.00	589 (1.0)	61,586 (1.0)	-0.00
Diabetes without chronic complication	1,466 (2.4)	149,341 (2.4)	0.00	1,557 (2.6)	149,275 (2.4)	0.01
Diabetes with chronic complication	158 (0.3)	23,562 (0.4)	-0.02	211 (0.3)	23,497 (0.4)	-0.01
Hemiplegia or paraplegia	34 (0.1)	3,170 (0.1)	0.00	43 (0.1)	3,166 (0.1)	0.01
HIV/AIDS	73 (0.1)	6,195 (0.1)	0.01	56 (0.1)	6,214 (0.1)	-0.00
Mild liver disease	96 (0.2)	8,657 (0.1)	0.01	84 (0.1)	8,664 (0.1)	-0.00
Severe liver disease	3 (0.0)	317 (0.0)	-0.00	4 (0.0)	318 (0.0)	0.00
Myocardial infarction	28 (0.0)	3,068 (0.0)	-0.00	32 (0.1)	3,067 (0.0)	0.00
Peptic ulcer	23 (0.0)	2,026 (0.0)	0.00	23 (0.0)	2,025 (0.0)	0.00
Peripheral artery disease	20 (0.0)	2,673 (0.0)	-0.01	26 (0.0)	2,668 (0.0)	0.00
Renal disease	11 (0.0)	3,399 (0.1)	-0.02	17 (0.0)	3,392 (0.1)	-0.01
Rheumatic disease	17 (0.0)	3,228 (0.1)	-0.01	24 (0.0)	3,224 (0.1)	-0.01
**Cardiometabolic comorbidities, n (%)**						
Atrial fibrillation	43 (0.1)	4,932 (0.1)	-0.00	49 (0.1)	4,931 (0.1)	0.00
Coronary artery disease	75 (0.1)	9,667 (0.2)	-0.01	86 (0.1)	9,658 (0.2)	-0.00
Diabetes mellitus	1,748 (2.9)	189,417 (3.1)	-0.01	1,914 (3.2)	189,262 (3.1)	0.00
Endocarditis	2 (0.0)	399 (0.0)	-0.00	1 (0.0)	400 (0.0)	-0.01
ESRD	2 (0.0)	807 (0.0)	-0.01	3 (0.0)	805 (0.0)	-0.01
Hyperlipidemia	1,378 (2.3)	136,788 (2.2)	0.01	1,366 (2.3)	136,793 (2.2)	0.00
Hypertension	505 (0.8)	66,642 (1.1)	-0.02	556 (0.9)	66,576 (1.1)	-0.02
Stroke	18 (0.0)	1,964 (0.0)	-0.00	22 (0.0)	1,962 (0.0)	0.00
Valvular heart disease	39 (0.1)	5,154 (0.1)	-0.01	43 (0.1)	5,153 (0.1)	-0.00
**Comorbidities associated with TdP, n (%)**						
AV block	0 (0.0)	93 (0.0)	-0.01	0 (0.0)	93 (0.0)	-0.01
Ankylosing spondylitis	0 (0.0)	127 (0.0)	-0.01	0 (0.0)	127 (0.0)	-0.01
Hyperparathyroidism	13 (0.0)	986 (0.0)	0.00	13 (0.0)	986 (0.0)	0.00
Hypothyroidism	1,249 (2.1)	128,844 (2.1)	-0.00	1,269 (2.1)	128,834 (2.1)	-0.00
Panhypopituitarism	5 (0.0)	267 (0.0)	0.01	4 (0.0)	267 (0.0)	0.00
Rheumatic arthritis	2 (0.0)	1,373 (0.0)	-0.02	3 (0.0)	1,370 (0.0)	-0.01
**Psychiatric comorbidities, n (%)**						

© 2026 Chien H et al. *JAMA Network Open*.

Covariates	Before weighting			After weighting		
	Initiators (n=59,971)	Non-initiators (n=6,159,116)	SMD	Initiators (n=60,695)	Non-initiators (n=6,159,110)	SMD
Alcohol use disorder	2,311 (3.9)	135,546 (2.2)	0.10	1,358 (2.2)	136,529 (2.2)	0.00
Anxiety	992 (1.7)	84,560 (1.4)	0.02	779 (1.3)	84,725 (1.4)	-0.01
Depression	12,476 (20.8)	926,748 (15.0)	0.15	8,083 (13.3)	930,138 (15.1)	-0.05
Schizophrenia	1,092 (1.8)	98,924 (1.6)	0.02	1,029 (1.7)	99,052 (1.6)	0.01
**Co-medications, n (%)**						
Medication with known risk of QT-prolongation	7,377 (12.3)	654,166 (10.6)	0.05	6,542 (10.8)	655,164 (10.6)	0.00
Medications as strong CYP inhibitors						
CYP1A2 inhibitors	890 (1.5)	74,758 (1.2)	0.02	719 (1.2)	74,918 (1.2)	-0.00
CYP2D6 inhibitors	4,410 (7.4)	617,268 (10.0)	-0.09	6,583 (10.8)	615,686 (10.0)	0.03
CYP3A4 inhibitors	191 (0.3)	18,207 (0.3)	0.00	181 (0.3)	18,221 (0.3)	0.00
Other antidepressants	14,313 (23.9)	2,117,052 (34.4)	-0.23	20,529 (33.8)	2,110,807 (34.3)	-0.01
**HCRU in prior 1 year, mean ± SD**						
OPD visits	16.9 ± 18.1	21.8 ± 20.0	-0.26	22.0 ± 20.6	21.8 ± 20.0	0.01
ED visits	1.1 ± 2.2	1.0 ± 2.1	0.06	1.1 ± 2.2	1.0 ± 2.1	0.03
Total hospitalization stays	4.9 ± 12.0	3.5 ± 9.8	0.12	3.9 ± 11.4	3.6 ± 9.8	0.03

(B) Taiwan cohort

Covariates	Before weighting			After weighting		
	Initiators (n=34,271)	Non-initiators (n=3,302,156)	SMD	Initiators (n=34,387)	Non-initiators (n=3,061,691)	SMD
**Age, mean ± SD**	45.8 ± 18.1	50.4 ± 18.2	-0.26	51.2 ± 18.7	50.6 ± 18.2	0.03
**Sex, n (%)**			0.07			0
Female	20,401 (59.5)	1,859,285 (56.3)		19,100 (55.5)	1,699,998 (55.5)	
Male	13,870 (40.5)	1,442,871 (43.7)		15,287 (44.5)	1,361,693 (44.5)	
**Antipsychotics, n (%)**			0.55			0.06
Amisulpride	696 (2.0)	78,566 (2.4)		926 (2.7)	77,496 (2.5)	
Aripiprazole	3,072 (9.0)	247,647 (7.5)		2,574 (7.5)	237,390 (7.8)	
Flupentixol	5,634 (16.4)	330,353 (10.0)		3,565 (10.4)	311,140 (10.2)	
Haloperidol	254 (0.7)	48,326 (1.5)		583 (1.7)	46,959 (1.5)	
Olanzapine	1,298 (3.8)	109,088 (3.3)		1,126 (3.3)	103,430 (3.4)	
Prochlorperazine	561 (1.6)	367,969 (11.1)		2,914 (8.5)	268,350 (8.8)	
Quetiapine	7,061 (20.6)	900,709 (27.3)		10,674 (31.0)	890,110 (29.1)	
Risperidone	2,441 (7.1)	345,310 (10.5)		3,487 (10.1)	339,768 (11.1)	
Sulpiride	12,381 (36.1)	739,814 (22.4)		6,953 (20.2)	655,169 (21.4)	
Other antipsychotics*	873 (2.5)	134,374 (4.1)		1,585 (4.6)	131,877 (4.3)	
**Clinical variables**						

© 2026 Chien H et al. *JAMA Network Open*.

Covariates	Before weighting			After weighting		
	Initiators (n=34,271)	Non-initiators (n=3,302,156)	SMD	Initiators (n=34,387)	Non-initiators (n=3,061,691)	SMD
CCI score, n (%)			0.18			0.07
0	23,447 (68.4)	1,988,323 (60.2)		19,650 (57.1)	1,848,101 (60.4)	
1	5,495 (16.0)	619,879 (18.8)		6,645 (19.3)	575,084 (18.8)	
2	2,713 (7.9)	334,452 (10.1)		3,782 (11.0)	309,383 (10.1)	
≥ 3	2,616 (7.6)	359,502 (10.9)		4,311 (12.5)	329,123 (10.7)	
**Comorbidities in CCI, n (%)**						
Cancer	1,162 (3.4)	143,527 (4.3)	-0.05	1,670 (4.9)	128,263 (4.2)	0.03
Metastatic cancer	144 (0.4)	20,476 (0.6)	-0.03	219 (0.6)	16,562 (0.5)	0.01
Cerebral vascular disease	1,631 (4.8)	215,240 (6.5)	-0.08	2,734 (7.9)	198,876 (6.5)	0.06
Chronic pulmonary disease	1,953 (5.7)	240,611 (7.3)	-0.06	2,659 (7.7)	221,902 (7.2)	0.02
Congestive heart failure	641 (1.9)	80,436 (2.4)	-0.04	949 (2.8)	74,917 (2.4)	0.02
Dementia	937 (2.7)	189,344 (5.7)	-0.15	2,300 (6.7)	179,743 (5.9)	0.03
Diabetes without chronic complication	3,043 (8.9)	385,116 (11.7)	-0.09	4,360 (12.7)	355,984 (11.6)	0.03
Diabetes with chronic complication	940 (2.7)	122,905 (3.7)	-0.06	1,294 (3.8)	112,884 (3.7)	0
Hemiplegia or paraplegia	172 (0.5)	22,289 (0.7)	-0.02	265 (0.8)	20,742 (0.7)	0.01
HIV/AIDS	120 (0.4)	14,672 (0.4)	-0.01	150 (0.4)	14,005 (0.5)	0
Mild liver disease	2,251 (6.6)	258,192 (7.8)	-0.05	2,924 (8.5)	238,580 (7.8)	0.03
Severe liver disease	58 (0.2)	7,391 (0.2)	-0.01	88 (0.3)	6,915 (0.2)	0.01
Myocardial infarction	141 (0.4)	15,080 (0.5)	-0.01	243 (0.7)	13,705 (0.4)	0.03
Peptic ulcer	3,028 (8.8)	380,486 (11.5)	-0.09	3,989 (11.6)	348,934 (11.4)	0.01
Peripheral artery disease	318 (0.9)	39,206 (1.2)	-0.03	481 (1.4)	35,831 (1.2)	0.02
Renal disease	882 (2.6)	112,602 (3.4)	-0.05	1,374 (4.0)	103,753 (3.4)	0.03
Rheumatic disease	720 (2.1)	78,809 (2.4)	-0.02	932 (2.7)	71,972 (2.4)	0.02
**Cardiometabolic comorbidities, n (%)**						
Atrial fibrillation	290 (0.8)	31,971 (1.0)	-0.01	369 (1.1)	29,705 (1.0)	0.01
Coronary artery disease	1,861 (5.4)	227,253 (6.9)	-0.06	2,658 (7.7)	210,254 (6.9)	0.03
Diabetes mellitus	3,309 (9.7)	420,997 (12.7)	-0.1	4,716 (13.7)	388,647 (12.7)	0.03
Endocarditis	29 (0.1)	4,255 (0.1)	-0.01	43 (0.1)	3,894 (0.1)	0
ESRD	241 (0.7)	30,273 (0.9)	-0.02	405 (1.2)	27,593 (0.9)	0.03
Hyperlipidemia	4,109 (12.0)	475,452 (14.4)	-0.07	5,300 (15.4)	439,479 (14.4)	0.03
Hypertension	6,633 (19.4)	829,934 (25.1)	-0.14	9,319 (27.1)	767,731 (25.1)	0.05
Stroke	641 (1.9)	85,332 (2.6)	-0.05	1,076 (3.1)	79,155 (2.6)	0.03
Valvular heart disease	359 (1.0)	41,255 (1.2)	-0.02	451 (1.3)	37,970 (1.2)	0.01
**Comorbidities associated with TdP, n (%)**						
AV block	17 (0.0)	1,057 (0.0)	0.01	22 (0.1)	949 (0.0)	0.02
Ankylosing spondylitis	68 (0.2)	9,070 (0.3)	-0.02	114 (0.3)	8,435 (0.3)	0.01

© 2026 Chien H et al. *JAMA Network Open*.

Covariates	Before weighting			After weighting		
	Initiators (n=34,271)	Non-initiators (n=3,302,156)	SMD	Initiators (n=34,387)	Non-initiators (n=3,061,691)	SMD
Hyperparathyroidism	28 (0.1)	2,291 (0.1)	0	39 (0.1)	2,164 (0.1)	0.01
Hypothyroidism	267 (0.8)	34,934 (1.1)	-0.03	322 (0.9)	32,285 (1.1)	-0.01
Panhypopituitarism	5 (0.0)	348 (0.0)	0	6 (0.0)	333 (0.0)	0
Rheumatic arthritis	205 (0.6)	24,223 (0.7)	-0.02	286 (0.8)	21,800 (0.7)	0.01
**Psychiatric comorbidities, n (%)**						
Alcohol use disorder	1,032 (3.0)	112,806 (3.4)	-0.02	1,463 (4.3)	107,032 (3.5)	0.04
Anxiety	14,799 (43.2)	1,143,091 (34.6)	0.18	13,123 (38.2)	1,078,505 (35.2)	0.06
Depression	27,165 (79.3)	1,625,174 (49.2)	0.66	16,970 (49.4)	1,499,946 (49.0)	0.01
Schizophrenia	1,808 (5.3)	399,701 (12.1)	-0.24	3,928 (11.4)	390,165 (12.7)	-0.04
**Co-medications, n (%)**						
Medication with known risk of QT-prolongation	5,386 (15.7)	553,862 (16.8)	-0.03	6,310 (18.3)	499,979 (16.3)	0.05
Medications as strong CYP inhibitors						
CYP1A2 inhibitors	277 (0.8)	31,915 (1.0)	-0.02	387 (1.1)	28,419 (0.9)	0.02
CYP2D6 inhibitors	1,544 (4.5)	149,797 (4.5)	0	2,366 (6.9)	143,866 (4.7)	0.09
CYP3A4 inhibitors	382 (1.1)	46,155 (1.4)	-0.03	511 (1.5)	41,851 (1.4)	0.01
Other antidepressants	8,868 (25.9)	1,159,555 (35.1)	-0.2	13,647 (39.7)	1,093,042 (35.7)	0.08
**HCRU in prior 1 year, mean ± SD**						
OPD visits	17.6 ± 15.8	21.5 ± 17.0	-0.24	22.8 ± 17.9	21.5 ± 17.0	0.08
ED visits	0.6 ± 1.2	0.6 ± 1.6	-0.04	0.7 ± 1.5	0.6 ± 1.6	0.07
Total hospitalization stays	1.9 ± 10.4	2.8 ± 13.9	-0.07	3.1 ± 13.9	2.8 ± 14.0	0.02

n represents the number of person-trials; individuals could contribute to multiple non-initiator trials until SSRI initiation or censoring.

*'Other antipsychotics' category includes the following drugs: brexpiprazole, chlorpromazine, chlorprothixene, clozapine, clothiapine, droperidol, fluphenazine, loxapine, lurasidone, paliperidone, perphenazine, pimozide, thioridazine, trifluoperazine, ziprasidone, and zotepine. These drugs were grouped together due to their relatively low frequency of use in the study population.

Abbreviations: AV block, atrioventricular block; CCI, Charlson comorbidity index; CYP, cytochrome P450; ED, emergency department; ESRD, end-stage renal disease; HCRU, healthcare resource utilization; HIV/AIDS, human immunodeficiency virus/acquired immunodeficiency syndrome; OPD, outpatient department; SD, standard deviation; SMD, standardized mean difference; SSRI, selective serotonin reuptake inhibitor; TdP, torsades de pointes.

© 2026 Chien H et al. *JAMA Network Open*.

eTable 4. Baseline characteristics of SNRI initiators and non-initiators at the start of trial follow-up: in the US cohort

Covariates	Before weighting			After weighting		
	Initiators (n=25,337)	Non-initiators (n=7,026,937)	SMD	Initiators (n=25,491)	Non-initiators (n=7,026,937)	SMD
**Age, mean ± SD**	42.2 ± 16.4	45.4 ± 19.4	-0.17	45.2 ± 18.2	45.3 ± 19.4	-0.01
**Sex, n (%)**			0.05			-0.00
Female	16,386 (64.7)	4,359,970 (62.0)		15,798 (62.0)	4,360,632 (62.1)	
Male	8,951 (35.3)	2,666,967 (38.0)		9,693 (38.0)	2,666,305 (37.9)	
**Antipsychotics, n (%)**			0.19			0.04
Aripiprazole	9,145 (36.1)	2,362,066 (33.6)		8,812 (34.6)	2,362,692 (33.6)	
Brexpiprazole	407 (1.6)	143,470 (2.0)		524 (2.1)	143,360 (2.0)	
Cariprazine	287 (1.1)	127,067 (1.8)		463 (1.8)	126,896 (1.8)	
Chlorpromazine	62 (0.2)	21,875 (0.3)		70 (0.3)	21,858 (0.3)	
Clozapine	17 (0.1)	6,650 (0.1)		23 (0.1)	6,643 (0.1)	
Droperidol	1 (0.0)	41 (0.0)		0 (0.0)	42 (0.0)	
Fluphenazine	8 (0.0)	6,629 (0.1)		19 (0.1)	6,613 (0.1)	
Haloperidol	160 (0.6)	80,540 (1.1)		281 (1.1)	80,410 (1.1)	
Iloperidone	5 (0.0)	3,175 (0.0)		11 (0.0)	3,169 (0.0)	
Loxapine	19 (0.1)	5,001 (0.1)		20 (0.1)	5,002 (0.1)	
Lumateperone	6 (0.0)	2,389 (0.0)		9 (0.0)	2,386 (0.0)	
Lurasidone	604 (2.4)	248,735 (3.5)		911 (3.6)	248,443 (3.5)	
Olanzapine	1,777 (7.0)	517,514 (7.4)		1,926 (7.6)	517,425 (7.4)	
Paliperidone	54 (0.2)	24,164 (0.3)		87 (0.3)	24,131 (0.3)	
Perphenazine	101 (0.4)	22,788 (0.3)		80 (0.3)	22,807 (0.3)	
Pimavanserin	2 (0.0)	4,345 (0.1)		13 (0.0)	4,331 (0.1)	
Pimozide	4 (0.0)	2,630 (0.0)		16 (0.1)	2,625 (0.0)	
Prochlorperazine	703 (2.8)	334,346 (4.8)		1,283 (5.0)	333,845 (4.8)	
Quetiapine	9,151 (36.1)	2,255,125 (32.1)		7,920 (31.1)	2,256,140 (32.1)	
Risperidone	2,326 (9.2)	734,614 (10.5)		2,581 (10.1)	734,292 (10.4)	
Thioridazine	1 (0.0)	2,596 (0.0)		4 (0.0)	2,588 (0.0)	
Trifluoperazine	12 (0.0)	4,048 (0.1)		11 (0.0)	4,045 (0.1)	
Ziprasidone	485 (1.9)	117,129 (1.7)		424 (1.7)	117,191 (1.7)	
**Clinical variables**						
CCI score, n (%)			0.06			0.02
0	24,068 (95.0)	6,581,723 (93.7)		23,944 (93.9)	6,582,058 (93.7)	
1	881 (3.5)	288,388 (4.1)		960 (3.8)	288,229 (4.1)	
2	366 (1.4)	146,582 (2.1)		552 (2.2)	146,420 (2.1)	
≥ 3	22 (0.1)	10,244 (0.1)		35 (0.1)	10,229 (0.1)	
**Comorbidities in CCI, n (%)**						

© 2026 Chien H et al. *JAMA Network Open*.

Covariates	Before weighting			After weighting		
	Initiators (n=25,337)	Non-initiators (n=7,026,937)	SMD	Initiators (n=25,491)	Non-initiators (n=7,026,937)	SMD
Cancer	272 (1.1)	116,108 (1.7)	-0.05	429 (1.7)	115,970 (1.7)	0.00
Metastatic cancer	7 (0.0)	3,622 (0.1)	-0.01	13 (0.1)	3,615 (0.1)	0.00
Cerebral vascular disease	27 (0.1)	9,251 (0.1)	-0.01	39 (0.2)	9,245 (0.1)	0.01
Chronic pulmonary disease	70 (0.3)	17,817 (0.3)	0.00	67 (0.3)	17,817 (0.3)	0.00
Congestive heart failure	15 (0.1)	5,982 (0.1)	-0.01	20 (0.1)	5,976 (0.1)	-0.00
Dementia	102 (0.4)	79,169 (1.1)	-0.08	163 (0.6)	79,064 (1.1)	-0.05
Diabetes without chronic complication	621 (2.5)	158,817 (2.3)	0.01	633 (2.5)	158,777 (2.3)	0.01
Diabetes with chronic complication	69 (0.3)	22,756 (0.3)	-0.01	94 (0.4)	22,738 (0.3)	0.01
Hemiplegia or paraplegia	13 (0.1)	3,839 (0.1)	-0.00	14 (0.1)	3,836 (0.1)	-0.00
HIV/AIDS	14 (0.1)	6,146 (0.1)	-0.01	20 (0.1)	6,139 (0.1)	-0.00
Mild liver disease	30 (0.1)	8,011 (0.1)	0.00	24 (0.1)	8,014 (0.1)	-0.01
Severe liver disease	1 (0.0)	341 (0.0)	-0.00	1 (0.0)	341 (0.0)	-0.00
Myocardial infarction	7 (0.0)	3,183 (0.0)	-0.01	8 (0.0)	3,181 (0.0)	-0.01
Peptic ulcer	3 (0.0)	2,239 (0.0)	-0.01	3 (0.0)	2,240 (0.0)	-0.01
Peripheral artery disease	5 (0.0)	2,632 (0.0)	-0.01	4 (0.0)	2,629 (0.0)	-0.01
Renal disease	8 (0.0)	3,589 (0.1)	-0.01	10 (0.0)	3,586 (0.1)	-0.01
Rheumatic disease	9 (0.0)	2,629 (0.0)	-0.00	9 (0.0)	2,629 (0.0)	-0.00
**Cardiometabolic comorbidities, n (%)**						
Atrial fibrillation	13 (0.1)	6,297 (0.1)	-0.01	15 (0.1)	6,292 (0.1)	-0.01
Coronary artery disease	27 (0.1)	10,986 (0.2)	-0.01	31 (0.1)	10,978 (0.2)	-0.01
Diabetes mellitus	746 (2.9)	197,597 (2.8)	0.01	781 (3.1)	197,541 (2.8)	0.02
Endocarditis	2 (0.0)	356 (0.0)	0.00	1 (0.0)	356 (0.0)	-0.00
ESRD	4 (0.0)	986 (0.0)	0.00	4 (0.0)	985 (0.0)	0.00
Hyperlipidemia	574 (2.3)	160,346 (2.3)	-0.00	530 (2.1)	160,375 (2.3)	-0.01
Hypertension	176 (0.7)	64,180 (0.9)	-0.02	155 (0.6)	64,162 (0.9)	-0.03
Stroke	7 (0.0)	2,348 (0.0)	-0.00	10 (0.0)	2,346 (0.0)	0.00
Valvular heart disease	12 (0.0)	5,616 (0.1)	-0.01	15 (0.1)	5,614 (0.1)	-0.01
**Comorbidities associated with TdP, n (%)**						
AV block	0 (0.0)	207 (0.0)	-0.01	0 (0.0)	207 (0.0)	-0.01
Ankylosing spondylitis	0 (0.0)	128 (0.0)	-0.01	0 (0.0)	128 (0.0)	-0.01
Hyperparathyroidism	4 (0.0)	976 (0.0)	0.00	3 (0.0)	976 (0.0)	-0.00
Hypothyroidism	557 (2.2)	144,931 (2.1)	0.01	526 (2.1)	144,964 (2.1)	0.00
Panhypopituitarism	1 (0.0)	333 (0.0)	-0.00	1 (0.0)	333 (0.0)	-0.00
Rheumatic arthritis	5 (0.0)	1,352 (0.0)	0.00	5 (0.0)	1,352 (0.0)	-0.00
**Psychiatric comorbidities, n (%)**						
Alcohol use disorder	905 (3.6)	173,523 (2.5)	0.06	636 (2.5)	173,801 (2.5)	0.00

© 2026 Chien H et al. *JAMA Network Open*.

Covariates	Before weighting			After weighting		
	Initiators (n=25,337)	Non-initiators (n=7,026,937)	SMD	Initiators (n=25,491)	Non-initiators (n=7,026,937)	SMD
Anxiety	477 (1.9)	119,564 (1.7)	0.01	432 (1.7)	119,610 (1.7)	-0.00
Depression	6,233 (24.6)	1,210,456 (17.2)	0.18	4,244 (16.6)	1,212,317 (17.3)	-0.02
Schizophrenia	136 (0.5)	80,124 (1.1)	-0.07	269 (1.1)	79,972 (1.1)	-0.01
**Co-medications, n (%)**						
Medication with known risk of QT-prolongation	3416 (13.5)	1,028,341 (14.6)	-0.03	3,514 (13.8)	1,028,049 (14.6)	-0.02
Medications as strong CYP inhibitors						
CYP1A2 inhibitors	438 (1.7)	120,610 (1.7)	0.00	434 (1.7)	120,613 (1.7)	-0.00
CYP2D6 inhibitors	3,136 (12.4)	975,078 (13.9)	-0.04	3,624 (14.2)	974,700 (13.9)	0.01
CYP3A4 inhibitors	97 (0.4)	24,793 (0.4)	0.00	86 (0.3)	24,801 (0.4)	-0.00
Other antidepressants	4,876 (19.2)	1,170,445 (16.7)	0.07	4,324 (17.0)	1,171,099 (16.7)	0.01
**HCRU in prior 1 year, mean ± SD**						
OPD visits	22.2 ± 19.7	21.5 ± 19.4	0.03	22.1 ± 19.7	21.5 ± 19.4	0.03
ED visits	1.2 ± 2.4	1.0 ± 2.0	0.09	1.0 ± 2.1	1.0 ± 2.1	-0.00
Total hospitalization stays	4.7 ± 11.7	3.4 ± 9.1	0.13	3.5 ± 10.1	3.4 ± 9.1	0.02

n represents the number of person-trials; individuals could contribute to multiple non-initiator trials until SSRI initiation or censoring.

Abbreviations: AV block, atrioventricular block; CCI, Charlson comorbidity index; CYP, cytochrome P450; ED, emergency department; ESRD, end-stage renal disease; HCRU, healthcare resource utilization; HIV/AIDS, human immunodeficiency virus/acquired immunodeficiency syndrome; OPD, outpatient department; SD, standard deviation; SMD, standardized mean difference; SNRI, serotonin-norepinephrine reuptake inhibitor; TdP, torsades de pointes.

© 2026 Chien H et al. *JAMA Network Open*.

eTable 5. Baseline characteristics of TCA initiators and non-initiators at the start of trial follow-up: (A) US cohort, (B) Taiwan cohort

(A) US cohort

Covariates	Before weighting			After weighting		
	Initiators (n=21,562)	Non-initiators (n=8,407,664)	SMD	Initiators (n=21,311)	Non-initiators (n=8,407,666)	SMD
**Age, mean ± SD**	47.0 ± 19.5	45.1 ± 18.2	0.10	45.5 ± 18.2	45.1 ± 18.2	0.02
**Sex, n (%)**			-0.06			-0.01
Female	13,365 (62.0)	5,437,260 (64.7)		13,689 (64.2)	5,436,683 (64.7)	
Male	8,197 (38.0)	2,970,404 (35.3)		7,622 (35.8)	2,970,983 (35.3)	
**Antipsychotics, n (%)**			0.34			0.04
Aripiprazole	5,992 (27.8)	3,231,688 (38.4)		7,898 (37.1)	3,229,397 (38.4)	
Brexpiprazole	278 (1.3)	205,328 (2.4)		531 (2.5)	205,080 (2.4)	
Cariprazine	222 (1.0)	151,445 (1.8)		387 (1.8)	151,279 (1.8)	
Chlorpromazine	127 (0.6)	21,255 (0.3)		57 (0.3)	21,327 (0.3)	
Clozapine	8 (0.0)	6,976 (0.1)		16 (0.1)	6,966 (0.1)	
Droperidol	1 (0.0)	23 (0.0)		0 (0.0)	24 (0.0)	
Fluphenazine	20 (0.1)	6,569 (0.1)		17 (0.1)	6,572 (0.1)	
Haloperidol	389 (1.8)	75,920 (0.9)		194 (0.9)	76,114 (0.9)	
Iloperidone	7 (0.0)	3,491 (0.0)		7 (0.0)	3,489 (0.0)	
Loxapine	21 (0.1)	5,223 (0.1)		14 (0.1)	5,231 (0.1)	
Lumateperone	7 (0.0)	2,814 (0.0)		6 (0.0)	2,814 (0.0)	
Lurasidone	488 (2.3)	297,552 (3.5)		710 (3.3)	297,278 (3.5)	
Olanzapine	1,818 (8.4)	544,150 (6.5)		1,389 (6.5)	544,571 (6.5)	
Paliperidone	47 (0.2)	25,552 (0.3)		67 (0.3)	25,534 (0.3)	
Perphenazine	367 (1.7)	19,166 (0.2)		59 (0.3)	19,485 (0.2)	
Pimavanserin	9 (0.0)	4,434 (0.1)		12 (0.1)	4,432 (0.1)	
Pimozide	4 (0.0)	3,050 (0.0)		8 (0.0)	3,046 (0.0)	
Prochlorperazine	1,460 (6.8)	361,608 (4.3)		983 (4.6)	362,140 (4.3)	
Quetiapine	7,394 (34.3)	2,520,912 (30.0)		6,592 (30.9)	2,521,839 (30.0)	
Risperidone	2,475 (11.5)	774,725 (9.2)		1,992 (9.3)	775,212 (9.2)	
Thioridazine	10 (0.0)	2,412 (0.0)		5 (0.0)	2,416 (0.0)	
Trifluoperazine	13 (0.1)	4,243 (0.1)		12 (0.1)	4,245 (0.1)	
Ziprasidone	405 (1.9)	139,128 (1.7)		355 (1.7)	139,176 (1.7)	
**Clinical variables**						
CCI score, n (%)			0.11			0.01
0	19,726 (91.5)	7,936,551 (94.4)		20,071 (94.2)	7,935,927 (94.4)	
1	1,239 (5.7)	305,247 (3.6)		804 (3.8)	305,702 (3.6)	
2	561 (2.6)	155,168 (1.8)		405 (1.9)	155,331 (1.8)	
≥ 3	36 (0.2)	10,698 (0.1)		31 (0.1)	10,707 (0.1)	

© 2026 Chien H et al. *JAMA Network Open*.

Covariates	Before weighting			After weighting		
	**Initiators** (n=21,562)	**Non-initiators** (n=8,407,664)	**SMD**	**Initiators** (n=21,311)	**Non-initiators** (n=8,407,666)	**SMD**
**Comorbidities in CCI, n (%)**						
Cancer	444 (2.1)	122,344 (1.5)	0.05	310 (1.5)	122,488 (1.5)	-0.00
Metastatic cancer	10 (0.0)	3,874 (0.0)	0.00	6 (0.0)	3,879 (0.0)	-0.01
Cerebral vascular disease	39 (0.2)	9,752 (0.1)	0.02	28 (0.1)	9,768 (0.1)	0.00
Chronic pulmonary disease	74 (0.3)	19,411 (0.2)	0.02	50 (0.2)	19,432 (0.2)	0.00
Congestive heart failure	17 (0.1)	5,760 (0.1)	0.00	11 (0.0)	5,772 (0.1)	-0.01
Dementia	390 (1.8)	69,508 (0.8)	0.09	232 (1.1)	69,648 (0.8)	0.03
Diabetes without chronic complication	637 (3.0)	182,277 (2.2)	0.05	424 (2.0)	182,516 (2.2)	-0.01
Diabetes with chronic complication	91 (0.4)	24,928 (0.3)	0.02	73 (0.3)	24,941 (0.3)	0.01
Hemiplegia or paraplegia	14 (0.1)	3,913 (0.0)	0.01	13 (0.1)	3,915 (0.0)	0.01
HIV/AIDS	25 (0.1)	6,327 (0.1)	0.01	24 (0.1)	6,330 (0.1)	0.01
Mild liver disease	30 (0.1)	8,406 (0.1)	0.01	20 (0.1)	8,419 (0.1)	-0.00
Severe liver disease	1 (0.0)	334 (0.0)	0.00	1 (0.0)	334 (0.0)	-0.00
Myocardial infarction	14 (0.1)	3,093 (0.0)	0.01	9 (0.0)	3,100 (0.0)	0.00
Peptic ulcer	11 (0.1)	2,569 (0.0)	0.01	8 (0.0)	2,573 (0.0)	0.00
Peripheral artery disease	12 (0.1)	2,769 (0.0)	0.01	7 (0.0)	2,772 (0.0)	0.00
Renal disease	10 (0.0)	3,732 (0.0)	0.00	9 (0.0)	3,735 (0.0)	-0.00
Rheumatic disease	19 (0.1)	3,125 (0.0)	0.02	17 (0.1)	3,127 (0.0)	0.02
**Cardiometabolic comorbidities, n (%)**						
Atrial fibrillation	21 (0.1)	6,312 (0.1)	0.01	14 (0.1)	6,324 (0.1)	-0.00
Coronary artery disease	53 (0.2)	10,664 (0.1)	0.03	31 (0.1)	10,684 (0.1)	0.00
Diabetes mellitus	783 (3.6)	226,562 (2.7)	0.05	536 (2.5)	226,841 (2.7)	-0.01
Endocarditis	2 (0.0)	381 (0.0)	0.01	1 (0.0)	382 (0.0)	0.00
ESRD	4 (0.0)	1,063 (0.0)	0.00	3 (0.0)	1,064 (0.0)	0.00
Hyperlipidemia	741 (3.4)	180,823 (2.2)	0.08	511 (2.4)	181,027 (2.2)	0.02
Hypertension	266 (1.2)	69,086 (0.8)	0.04	185 (0.9)	69,177 (0.8)	0.00
Stroke	11 (0.1)	2,430 (0.0)	0.01	9 (0.0)	2,433 (0.0)	0.01
Valvular heart disease	34 (0.2)	5,660 (0.1)	0.03	22 (0.1)	5,670 (0.1)	0.01
**Comorbidities associated with TdP, n (%)**						
AV block	0 (0.0)	194 (0.0)	-0.01	0 (0.0)	194 (0.0)	-0.01
Ankylosing spondylitis	2 (0.0)	166 (0.0)	0.01	2 (0.0)	166 (0.0)	0.01
Hyperparathyroidism	3 (0.0)	874 (0.0)	0.00	2 (0.0)	875 (0.0)	-0.00
Hypothyroidism	645 (3.0)	170,691 (2.0)	0.06	432 (2.0)	170,904 (2.0)	-0.00
Panhypopituitarism	3 (0.0)	353 (0.0)	0.01	2 (0.0)	353 (0.0)	0.00
Rheumatic arthritis	9 (0.0)	1,519 (0.0)	0.01	7 (0.0)	1,520 (0.0)	0.01
**Psychiatric comorbidities, n (%)**						

© 2026 Chien H et al. *JAMA Network Open*.

Covariates	Before weighting			After weighting		
	Initiators (n=21,562)	Non-initiators (n=8,407,664)	SMD	Initiators (n=21,311)	Non-initiators (n=8,407,666)	SMD
Alcohol use disorder	1,021 (4.7)	183,502 (2.2)	0.14	515 (2.4)	184,052 (2.2)	0.02
Anxiety	442 (2.0)	135,007 (1.6)	0.03	330 (1.6)	135,102 (1.6)	-0.00
Depression	4,263 (19.8)	1,524,773 (18.1)	0.04	3,556 (16.7)	1,525,124 (18.1)	-0.04
Schizophrenia	196 (0.9)	79,193 (0.9)	-0.00	213 (1.0)	79,186 (0.9)	0.01
**Co-medications, n (%)**						
Medication with known risk of QT-prolongation	3,487 (16.2)	1,046,103 (12.4)	0.11	2,713 (12.7)	1,046,906 (12.5)	0.01
Medications as strong CYP inhibitors						
CYP1A2 inhibitors	442 (2.0)	121,940 (1.5)	0.05	316 (1.5)	122,069 (1.5)	0.00
CYP2D6 inhibitors	1,886 (8.7)	1,054,313 (12.5)	-0.12	2,669 (12.5)	1,053,497 (12.5)	-0.00
CYP3A4 inhibitors	75 (0.3)	25,694 (0.3)	0.01	62 (0.3)	25,703 (0.3)	-0.00
Other antidepressants	5,840 (27.1)	2,157,122 (25.7)	0.03	5,653 (26.5)	2,157,431 (25.7)	0.02
**HCRU in prior 1 year, mean ± SD**						
OPD visits	23.2 ± 20.4	22.2 ± 19.5	0.05	23.2 ± 19.9	22.2 ± 19.5	0.05
ED visits	1.6 ± 2.6	0.9 ± 1.9	0.28	1.0 ± 2.0	0.9 ± 1.9	0.03
Total hospitalization stays	6.6 ± 13.7	3.0 ± 8.5	0.32	3.4 ± 9.7	3.0 ± 8.5	0.05

(B) Taiwan cohort

Covariates	Before weighting			After weighting		
	Initiators (n=10,335)	Non-initiators (n=2,494,846)	SMD	Initiators (n=9,785)	Non-initiators (n=2,275,854)	SMD
**Age, mean ± SD**	49.4 ± 17.8	49.9 ± 18.5	-0.03	49.2 ± 18.1	49.5 ± 18.4	-0.02
**Sex, n (%)**			0			-0.04
Female	5,981 (57.8)	1,446,551 (58.0)		5,590 (57.1)	1,343,992 (59.1)	
Male	4,354 (42.2)	1,048,295 (42.0)		4,195 (42.9)	931,862 (40.9)	
**Antipsychotics, n (%)**			0.23			0.12
Amisulpride	176 (1.7)	56,647 (2.3)		213 (2.2)	52,016 (2.3)	
Aripiprazole	842 (8.1)	249,400 (10.0)		852 (8.7)	228,061 (10.0)	
Flupentixol	1,305 (12.6)	235,836 (9.5)		876 (9.0)	217,762 (9.6)	
Haloperidol	123 (1.2)	31,209 (1.3)		118 (1.2)	28,438 (1.2)	
Olanzapine	380 (3.7)	87,439 (3.5)		405 (4.1)	78,273 (3.4)	
Prochlorperazine	418 (4.0)	169,458 (6.8)		574 (5.9)	158,746 (7.0)	
Quetiapine	3,245 (31.3)	766,415 (30.7)		3,378 (34.5)	689,736 (30.3)	
Risperidone	633 (6.1)	242,934 (9.7)		776 (7.9)	211,624 (9.3)	
Sulpiride	2,781 (26.9)	561,209 (22.5)		2,210 (22.6)	528,823 (23.2)	
Other antipsychotics*	452 (4.4)	94,299 (3.8)		382 (3.9)	82,375 (3.6)	
**Clinical variables**						

© 2026 Chien H et al. *JAMA Network Open*.

Covariates	Before weighting			After weighting		
	Initiators (n=10,335)	Non-initiators (n=2,494,846)	SMD	Initiators (n=9,785)	Non-initiators (n=2,275,854)	SMD
CCI score, n (%)			0.03			0.04
0	6,302 (60.9)	1,531,776 (61.4)		5,986 (61.2)	1,418,177 (62.3)	
1	1,870 (18.1)	465,451 (18.7)		1,777 (18.2)	421,379 (18.5)	
2	1,059 (10.2)	245,436 (9.8)		967 (9.9)	217,233 (9.5)	
≥ 3	1,124 (10.9)	252,183 (10.1)		1,054 (10.8)	219,065 (9.6)	
**Comorbidities in CCI, n (%)**						
Cancer	543 (5.2)	97,660 (3.9)	0.06	461 (4.7)	88,039 (3.9)	0.04
Metastatic cancer	94 (0.9)	12,682 (0.5)	0.05	70 (0.7)	11,561 (0.5)	0.03
Cerebral vascular disease	561 (5.4)	164,371 (6.6)	-0.05	583 (6.0)	143,228 (6.3)	-0.01
Chronic pulmonary disease	742 (7.2)	177,858 (7.1)	0	758 (7.7)	155,336 (6.8)	0.04
Congestive heart failure	262 (2.5)	57,656 (2.3)	0.01	186 (1.9)	47,777 (2.1)	-0.01
Dementia	418 (4.0)	168,266 (6.7)	-0.12	567 (5.8)	134,175 (5.9)	0
Diabetes without chronic complication	1,162 (11.2)	274,654 (11.0)	0.01	1,112 (11.4)	246,208 (10.8)	0.02
Diabetes with chronic complication	375 (3.6)	84,030 (3.4)	0.01	324 (3.3)	74,480 (3.3)	0
Hemiplegia or paraplegia	57 (0.6)	17,809 (0.7)	-0.02	65 (0.7)	15,252 (0.7)	0
HIV/AIDS	50 (0.5)	9,596 (0.4)	0.01	48 (0.5)	8,674 (0.4)	0.02
Mild liver disease	910 (8.8)	181,256 (7.3)	0.06	789 (8.1)	162,233 (7.1)	0.04
Severe liver disease	36 (0.3)	4,047 (0.2)	0.04	18 (0.2)	3,441 (0.2)	0.01
Myocardial infarction	51 (0.5)	11,147 (0.4)	0.01	49 (0.5)	9,594 (0.4)	0.01
Peptic ulcer	1,152 (11.1)	261,875 (10.5)	0.02	1,080 (11.0)	237,747 (10.4)	0.02
Peripheral artery disease	114 (1.1)	26,831 (1.1)	0	121 (1.2)	23,945 (1.1)	0.02
Renal disease	356 (3.4)	77,624 (3.1)	0.02	320 (3.3)	68,421 (3.0)	0.02
Rheumatic disease	266 (2.6)	56,424 (2.3)	0.02	232 (2.4)	51,412 (2.3)	0.01
**Cardiometabolic comorbidities, n (%)**						
Atrial fibrillation	84 (0.8)	25,156 (1.0)	-0.02	75 (0.8)	21,777 (1.0)	-0.02
Coronary artery disease	727 (7.0)	165,715 (6.6)	0.02	699 (7.1)	147,167 (6.5)	0.03
Diabetes mellitus	1,264 (12.2)	300,407 (12.0)	0.01	1,199 (12.3)	269,492 (11.8)	0.01
Endocarditis	12 (0.1)	2,846 (0.1)	0	14 (0.1)	2,280 (0.1)	0.01
ESRD	87 (0.8)	20,708 (0.8)	0	67 (0.7)	17,841 (0.8)	-0.01
Hyperlipidemia	1,493 (14.4)	348,589 (14.0)	0.01	1,489 (15.2)	317,826 (14.0)	0.04
Hypertension	2,530 (24.4)	605,320 (24.3)	0	2,434 (24.9)	541,239 (23.8)	0.03
Stroke	229 (2.2)	66,558 (2.7)	-0.03	240 (2.5)	57,975 (2.5)	-0.01
Valvular heart disease	150 (1.4)	31,133 (1.2)	0.02	138 (1.4)	27,595 (1.2)	0.02
**Comorbidities associated with TdP, n (%)**						
AV block	4 (0.0)	943 (0.0)	0	7 (0.1)	856 (0.0)	0.01
Ankylosing spondylitis	29 (0.3)	6,545 (0.3)	0	26 (0.3)	5,853 (0.3)	0

© 2026 Chien H et al. *JAMA Network Open*.

Covariates	Before weighting			After weighting		
	Initiators (n=10,335)	Non-initiators (n=2,494,846)	SMD	Initiators (n=9,785)	Non-initiators (n=2,275,854)	SMD
Hyperparathyroidism	7 (0.1)	1,715 (0.1)	0	11 (0.1)	1,541 (0.1)	0.01
Hypothyroidism	119 (1.1)	27,303 (1.1)	0.01	107 (1.1)	24,277 (1.1)	0
Panhypopituitarism	0 (0.0)	290 (0.0)	-0.02	0 (0.0)	241 (0.0)	-0.01
Rheumatic arthritis	87 (0.8)	16,727 (0.7)	0.02	64 (0.7)	14,741 (0.6)	0
**Psychiatric comorbidities, n (%)**						
Alcohol use disorder	568 (5.5)	75,820 (3.0)	0.12	400 (4.1)	67,304 (3.0)	0.06
Anxiety	4,839 (46.7)	979,646 (39.3)	0.15	4,523 (46.2)	915,892 (40.2)	0.12
Depression	7,875 (76.1)	1,496,856 (60.0)	0.35	6,347 (64.9)	1,397,938 (61.4)	0.07
Schizophrenia	566 (5.5)	282,886 (11.3)	-0.21	863 (8.8)	215,830 (9.5)	-0.02
**Co-medications, n (%)**						
Medication with known risk of QT-prolongation	1,896 (18.3)	394,151 (15.8)	0.07	1,575 (16.1)	357,694 (15.7)	0.01
Medications as strong CYP inhibitors						
CYP1A2 inhibitors	108 (1.0)	20,662 (0.8)	0.02	81 (0.8)	17,766 (0.8)	0
CYP2D6 inhibitors	556 (5.4)	129,287 (5.2)	0.01	564 (5.8)	121,587 (5.3)	0.02
CYP3A4 inhibitors	129 (1.2)	32,226 (1.3)	0	145 (1.5)	29,099 (1.3)	0.02
Other antidepressants	3,385 (32.7)	721,784 (28.9)	0.08	3,344 (34.2)	672,248 (29.5)	0.1
**HCRU in prior 1 year, mean ± SD**						
OPD visits	21.7 ± 17.3	22.0 ± 16.3	-0.02	23.6 ± 18.2	21.7 ± 16.1	0.11
ED visits	0.7 ± 1.6	0.6 ± 1.3	0.1	0.7 ± 1.4	0.6 ± 1.2	0.08
Total hospitalization stays	2.6 ± 10.2	2.9 ± 15.0	-0.03	2.5 ± 9.0	2.6 ± 13.7	-0.01

n represents the number of person-trials; individuals could contribute to multiple non-initiator trials until SSRI initiation or censoring.

*'Other antipsychotics' category includes the following drugs: brexpiprazole, chlorpromazine, chlorprothixene, clozapine, clothiapine, droperidol, fluphenazine, loxapine, lurasidone, paliperidone, perphenazine, pimozide, thioridazine, trifluoperazine, ziprasidone, and zotepine. These drugs were grouped together due to their relatively low frequency of use in the study population.

Abbreviations: AV block, atrioventricular block; CCI, Charlson comorbidity index; CYP, cytochrome P450; ED, emergency department; ESRD, end-stage renal disease; HCRU, healthcare resource utilization; HIV/AIDS, human immunodeficiency virus/acquired immunodeficiency syndrome; OPD, outpatient department; SD, standard deviation; SMD, standardized mean difference; TCA, tricyclic antidepressant; TdP, torsades de pointes.

© 2026 Chien H et al. *JAMA Network Open*.

eTable 6. Study antipsychotics: TdP risk classification, CYP450 substrates, and availability in Taiwan NHIRD and the US MarketScan databases

Antipsychotics	Generation	NHIRD	MarketScan	TdP risk category	CYP enzyme substrates
Amisulpiride	SGA	V		Conditional	
Aripiprazole	SGA	V	V	Possible	2D6, 3A4/5
Brexpiprazole	SGA	V	V	No QT risk assigned†	2D6, 3A4/5
Cariprazine	SGA		V	No QT risk assigned†	2D6
Chlorpromazine	FGA	V	V	Known	2D6
Chlorprothixene	FGA	V		Known	
Clozapine	SGA	V	V	Possible	1A2
Clothiapine	FGA	V		Possible	
Droperidol	FGA	V	V	Known	
Flupentixol	FGA	V		Possible	
Fluphenazine	FGA	V	V	No QT risk assigned†	
Haloperidol	FGA	V	V	Known	1A2, 2D6, 3A4/5
Iloperidone	SGA		V	Possible	
Loxapine	FGA	V	V	No QT risk assigned†	
Lumateperone	SGA		V	Possible	
Lurasidone	SGA	V	V	Possible	
Olanzapine	SGA	V	V	Conditional	1A2
Paliperidone	SGA	V	V	Possible	
Perphenazine	FGA	V	V	Possible	2D6
Pimavanserin	SGA		V	Possible	2D6
Pimozide	FGA	V	V	Known	3A4/5
Prochlorperazine	FGA	V	V	No QT risk assigned†	
Quetiapine	SGA	V	V	Conditional	3A4/5
Risperidone	SGA	V	V	Conditional	2D6, 3A4/5
Sulpiride	SGA	V		Known	
Thioridazine	FGA	V	V	Known	2D6
Trifluoperazine	FGA	V	V	No QT risk assigned†	
Ziprasidone	SGA	V	V	Conditional	3A4/5
Zotepine	SGA	V		Possible	

Generation refers to first- (FGA) or second-generation (SGA) antipsychotics. TdP risk categories follow the *CredibleMeds* classification: **Known-risk**—drug prolongs the QT interval and is clearly linked to TdP at recommended doses; **Possible-risk**—drug can prolong the QT interval but, to date, lacks evidence of TdP at recommended doses; **Conditional-risk**—drug is associated with TdP only under specific circumstances (e.g., supratherapeutic dose, electrolyte imbalance, or interacting medications).

© 2026 Chien H et al. *JAMA Network Open*.

†'No QT risk assigned' indicates that *CredibleMeds* had not classified the drug at the time of review; the absence of a designation should not be interpreted as proof of safety with respect to QT-interval prolongation or TdP. 'CYP enzyme substrates' lists the principal cytochrome P450 isoenzymes responsible for the metabolism of the drug.

Abbreviations: CYP, cytochrome P450; FGA, first-generation antipsychotic; MarketScan, Merative™ MarketScan® Research Databases; NHIRD, National Health Insurance Research Database (Taiwan); SGA, second-generation antipsychotic; TdP, torsades de pointes.

© 2026 Chien H et al. *JAMA Network Open*.

eTable 7. Study antidepressants: therapeutic class, TdP risk classification, CYP450 inhibition profile, and availability in Taiwan NHIRD and the US MarketScan databases

Antidepressants	Class	NHIRD	MarketScan	TdP risk category	CYP enzyme inhibitor†
Citalopram	SSRI	V	V	Known	Weak 2D6
Escitalopram	SSRI	V	V	Known	Weak 2D6
Fluoxetine	SSRI	V	V	Conditional	Moderate 2C19, Strong 2D6
Fluvoxamine	SSRI	V	V	Conditional	Strong 1A2, Strong 2C19
Paroxetine	SSRI	V	V	Conditional	Strong 2D6
Sertraline	SSRI	V	V	Conditional	Weak 2D6
Vilazodone	SSRI		V	No QT risk assigned†	
Vortioxetine	SSRI	V	V	No QT risk assigned†	
Desvenlafaxine	SNRI		V	Possible	
Duloxetine	SNRI	V	V	No QT risk assigned†	Moderate 2D6
Levomilnacipran	SNRI		V	No QT risk assigned†	
Milnacipran	SNRI	V	V	No QT risk assigned†	
Venlafaxine	SNRI	V	V	Possible	
Viloxazine	SNRI		V	No QT risk assigned†	
Amitriptyline	TCA	V	V	Conditional	
Amoxapine	TCA		V	No QT risk assigned†	
Clomipramine	TCA	V	V	Conditional	Weak 2D6
Desipramine	TCA		V	Possible	
Doxepin	TCA	V	V	Conditional	Moderate 2D6
Imipramine	TCA	V	V	Possible	
Maprotiline	TCA	V	V	Possible	
Melitracen	TCA	V		No QT risk assigned†	
Mianserin	TCA	V		Possible	
Mirtazapine	TCA	V	V	Possible	
Nortriptyline	TCA		V	Possible	
Protriptyline	TCA		V	No QT risk assigned†	
Trimipramine	TCA		V	Possible	

Antidepressants are grouped as selective serotonin reuptake inhibitors (SSRIs) or tricyclic antidepressants (TCAs). TdP risk categories follow the *CredibleMeds* classification: **Known-risk**—the drug prolongs the QT interval and is clearly linked to TdP at recommended doses; **Possible-risk**—the drug can prolong the QT interval but, to date, lacks evidence of TdP when used as recommended; **Conditional-risk**—the drug is associated with TdP only under specific conditions (e.g., supratherapeutic dose, electrolyte disturbance, or interacting medicines).

†'No QT risk assigned' indicates that *CredibleMeds* had not classified the drug at the time of review; absence of assignment does **not** imply absence of risk. 'CYP enzyme inhibitor' denotes the principal cytochrome P450 isoenzyme(s) inhibited by the antidepressant and the approximate inhibition strength, adapted from FDA guidance: **Strong**—≥ 5-fold increase in the area under the curve (AUC) or ≥ 80 % decrease in clearance of a sensitive substrate; **Moderate**—≥ 2-fold increase in AUC

© 2026 Chien H et al. *JAMA Network Open*.

or 50–80 % decrease in clearance; **Weak**—≥ 1.25-fold but < 2-fold increase in AUC or 20–50 % decrease in clearance of a sensitive substrate.

Abbreviations: CYP, cytochrome P450; MarketScan, Merative™ MarketScan® Research Databases; NHIRD, National Health Insurance Research Database (Taiwan); SSRI, selective serotonin reuptake inhibitor; SNRI, serotonin-norepinephrine reuptake inhibitor; TCA, tricyclic antidepressant; TdP, torsades de pointes.

© 2026 Chien H et al. *JAMA Network Open*.
