# Supplementary material for: Ventricular Arrhythmia and Sudden Death Risk With Concomitant Antipsychotic and SSRI Use
Source: JAMA Netw Open. 2026 Apr 9;9(4):e266028. doi: 10.1001/jamanetworkopen.2026.6028 (PMC13067012; doi:10.1001/jamanetworkopen.2026.6028)
Supplement: Supplement 2. — Data Sharing Statement [file jamanetwopen-e266028-s002.pdf]

Data Sharing Statement

Chien. Ventricular Arrhythmia and Sudden Death Risk With Concomitant Antipsychotic and SSRI Use. *JAMA Netw Open*. Published April 09, 2026.
doi:10.1001/jamanetworkopen.2026.6028

Data
Data available: No

Additional Information
Explanation for why data not available: The data used in this study are subject to ethical and legal restrictions. The de-identified datasets from Taiwan are regulated by the Ministry of Health and Welfare and cannot be shared publicly. The MarketScan data are available from Merative under license to the Harvard Pilgrim Health Care Institute, and are likewise not publicly available.
